# Supplementary material for: BCAA insufficiency leads to premature ovarian insufficiency via ceramide‐induced elevation of ROS
Source: EMBO Mol Med. 2023 Feb 27;15(4):e17450. doi: 10.15252/emmm.202317450 (PMC10086587; doi:10.15252/emmm.202317450)
Supplement: Supplementary file 1 — Appendix [file EMMM-15-e17450-s009.pdf]

Appendix Figures

Appendix FigureS1 2
Appendix FigureS2 3
Appendix FigureS3 4
Appendix FigureS4 5
Appendix FigureS5 5

Appendix Figure

A

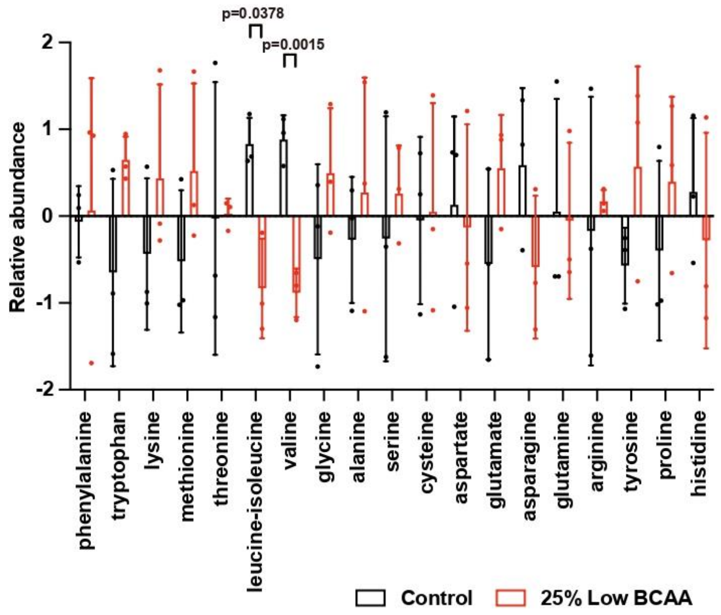

B

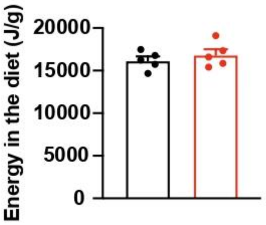

C

Ingredient (g)	Control	50% BCAA	25%BCAA
Amino acid mixture without BCAA	151	151	151
BCAA mixture	28	14	7
Corn starch	417	431	437
Dextrin and sucrose	250	250	250
Soy oil	50	50	50
Cellulose	50	50	50
Mineral mixture	43	43	43
Vitamin mixture	10	10	10
Choline chloride	2	2	2
TBHQ, antioxidant	0.01	0.01	0.01
Total	1000	1000	1000

D

Ingredient (g)	Control	25%BCAA
L-Arginine	10	10
L-Histidine-HCl-H2O	6	6
L-isoleucine	8	2
L-Leucine	12	2
L-Lysine-HCl	14	14
L-Methionine	6	6
L-Phenylalanine	8	8
L-Threonine	8	8
L-Typtophan	2	2
L-Valine	8	2
L-Alanine	10	10
L-Asparagine-H2O	5	5
L-Aspartate	10	10
L-Cystine	4	4
L-Glutamic Acid	30	30
L-Glutamine	5	5
Glycine	10	10
L-Proline	5	5
L-Serine	5	5
L-Tyrosine	4	4
Corn Starch	550.5	572.5
Maltodextrin 10	125	125
Cellulose	50	50
Corn Oil	50	50
Mineral Mix S10001	35	35
Sodium Bicarbonate	7.5	7.5
Vitamin Mix V10001	10	10
Choline Bitrartrate	2	2
FD&C Blue Dye #1	0.05	0.05
Total	1000.05	1000.05

Appendix FigureS1. Low BCAA diet on young female mice.

A. The relative abundance of amino acid composition of the low BCAA diet. N=3; **B.** The energy in the diet. N=5; and **C.** The recipe of the diet from Trophic Animal Feed High-tech; **D.** The recipe of the diet from Research Diet, Inc. Error bars stand for SEM of biological repeats. The p value was calculated by two-tailed t-test with 2-way ANOVA correction.

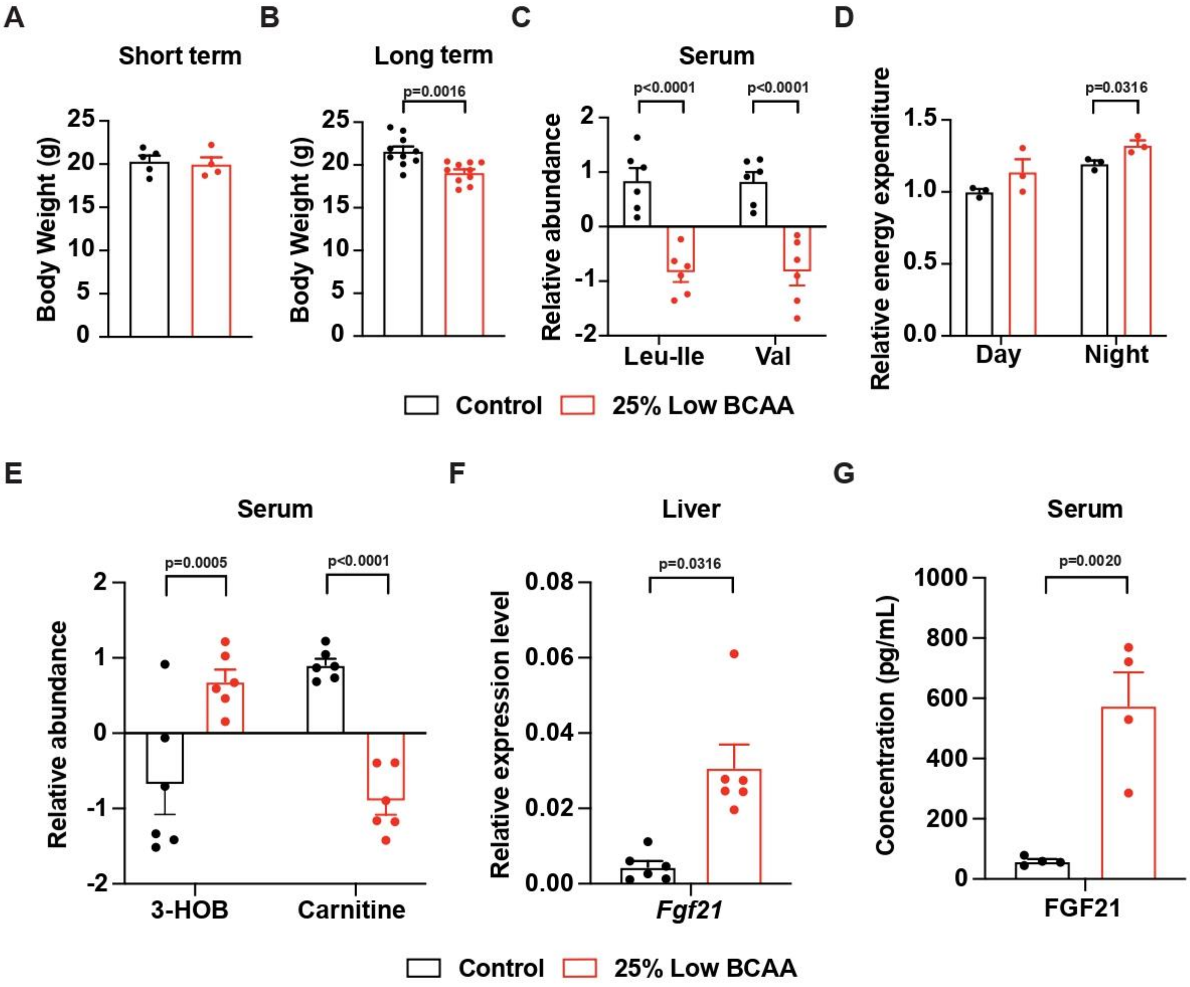

Appendix FigureS2. Metabolic features of young female mice fed a low BCAA diet.

A and B. The body weight of short (1.5 months; Control, N=5; Low BCAA, N=4) and long (3 months, N=10) term low BCAA diet; **C.** The relative abundance of BCAAs in serum. N=6; **D.** The relative energy expenditure measured by Comprehensive Lab Animal Monitoring System (CLAMS). N=3; **E.** The relative abundance of 3-hydroxybutyrate (3-HOB) and carnitine in serum. N=6; **F.** The mRNA level of Fgf21 in liver. N=6; **G.** The concentration of FGF21 in serum. N=4. Error bars stand for SEM of biological repeats. The p value was calculated by two-tailed t-test with 2-way ANOVA correction.

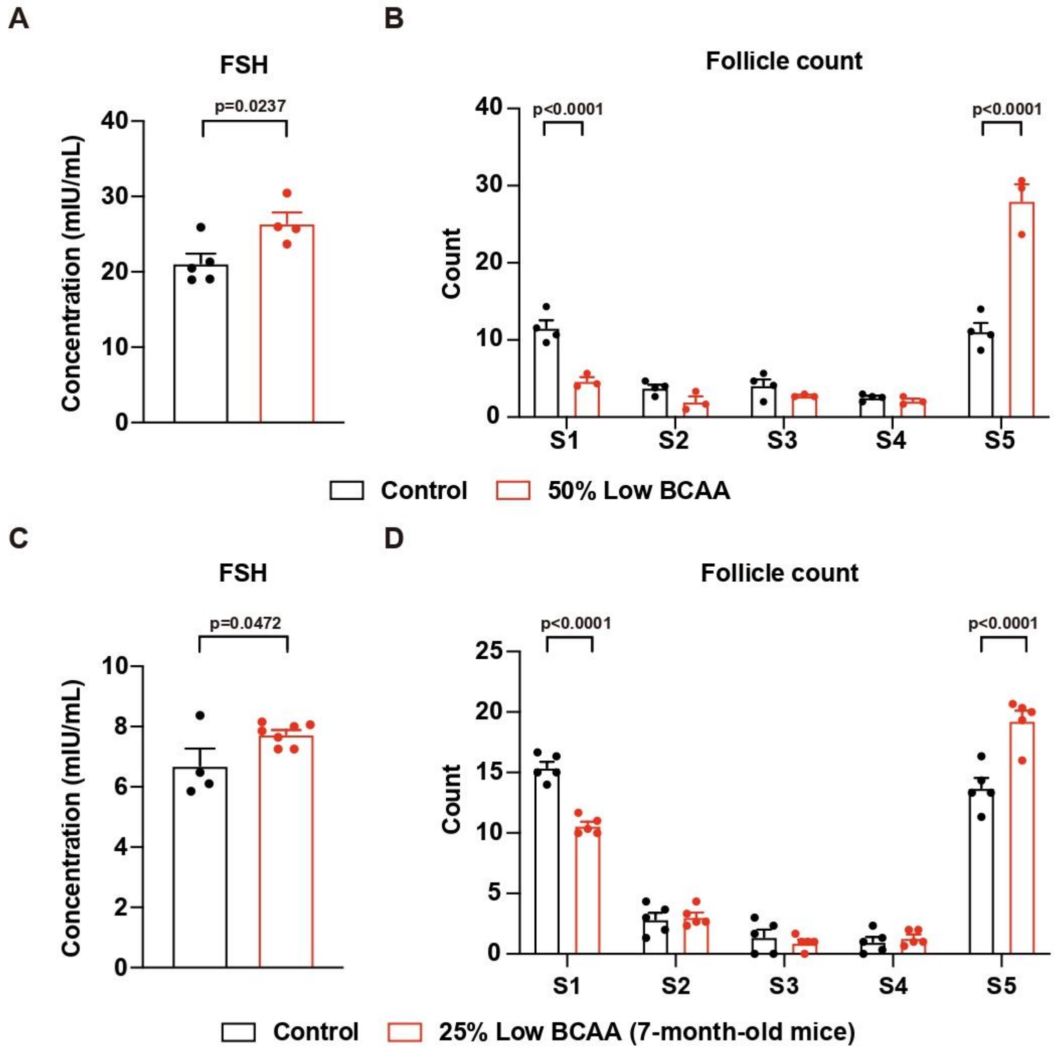

Appendix FigureS3. BCAA insufficiencies induce POI.

A. The serum concentration of FSH. Control, N=5; Low BCAA, N=4; and **B.** The changes of follicles in the mice on 50% low BCAA diet. Control, N=4; Low BCAA, N=3; **C.** Serum concentration of FSH. Control, N=4; Low BCAA, N=7; and **D.** The changes of follicles in 7-month-old mice on 25% low BCAA diet. N=5. S1, Primordial; S2, Primary; S3, Secondary; S4, Antral; S5, Atretic. Error bars stand for SEM of biological repeats. The p value was calculated by two-tailed t-test with 2-way ANOVA correction.

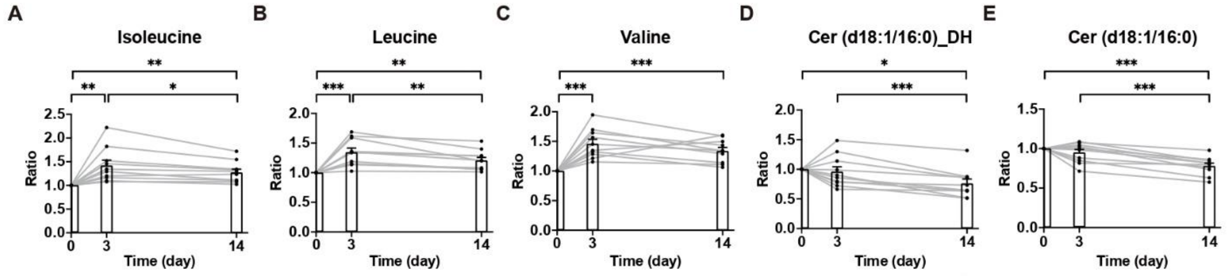

Appendix FigureS4. Relative abundance of metabolites in the serum of patients on a low carbohydrate high protein diet.

A. Serum concentration of isoleucine. N=7; **B.** Serum concentration of leucine. N=7; **C.** Serum concentration of valine. N=7; **D.** Serum concentration of cer (d18:1/16:0)_DH. N=7; **E.** Serum concentration of cer (d18:1/16:0). N=7. Error bars stand for SEM of biological repeats. The p value was calculated by two-tailed t-test with 2-way ANOVA correction.

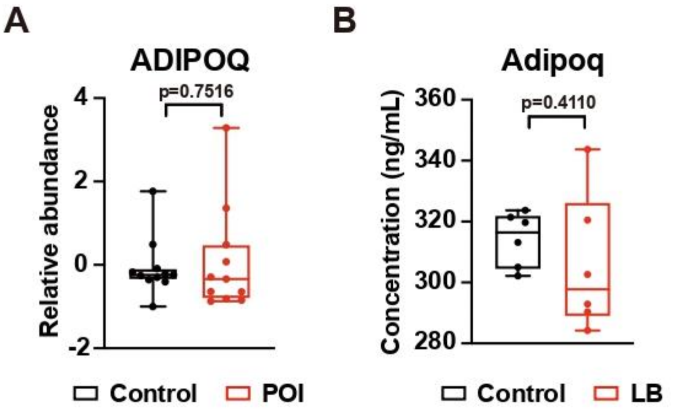

Appendix FigureS5. The changes of adiponectin in POI patients and mice on a low BCAA diet.

A. Serum concentration of adiponectin in POI patients and healthy donors. N=18; **B.** Serum concentration of adiponectin in mice fed by low BCAA diet (LB) or control diet. N=6. Error bars stand for SEM of biological repeats.
