# Supplementary material for: BCAA insufficiency leads to premature ovarian insufficiency via ceramide‐induced elevation of ROS
Source: EMBO Mol Med. 2023 Feb 27;15(4):e17450. doi: 10.15252/emmm.202317450 (PMC10086587; doi:10.15252/emmm.202317450)
Supplement: Supplementary file 2 — Expanded View Figures PDF [file EMMM-15-e17450-s017.pdf]

Expanded View Figures

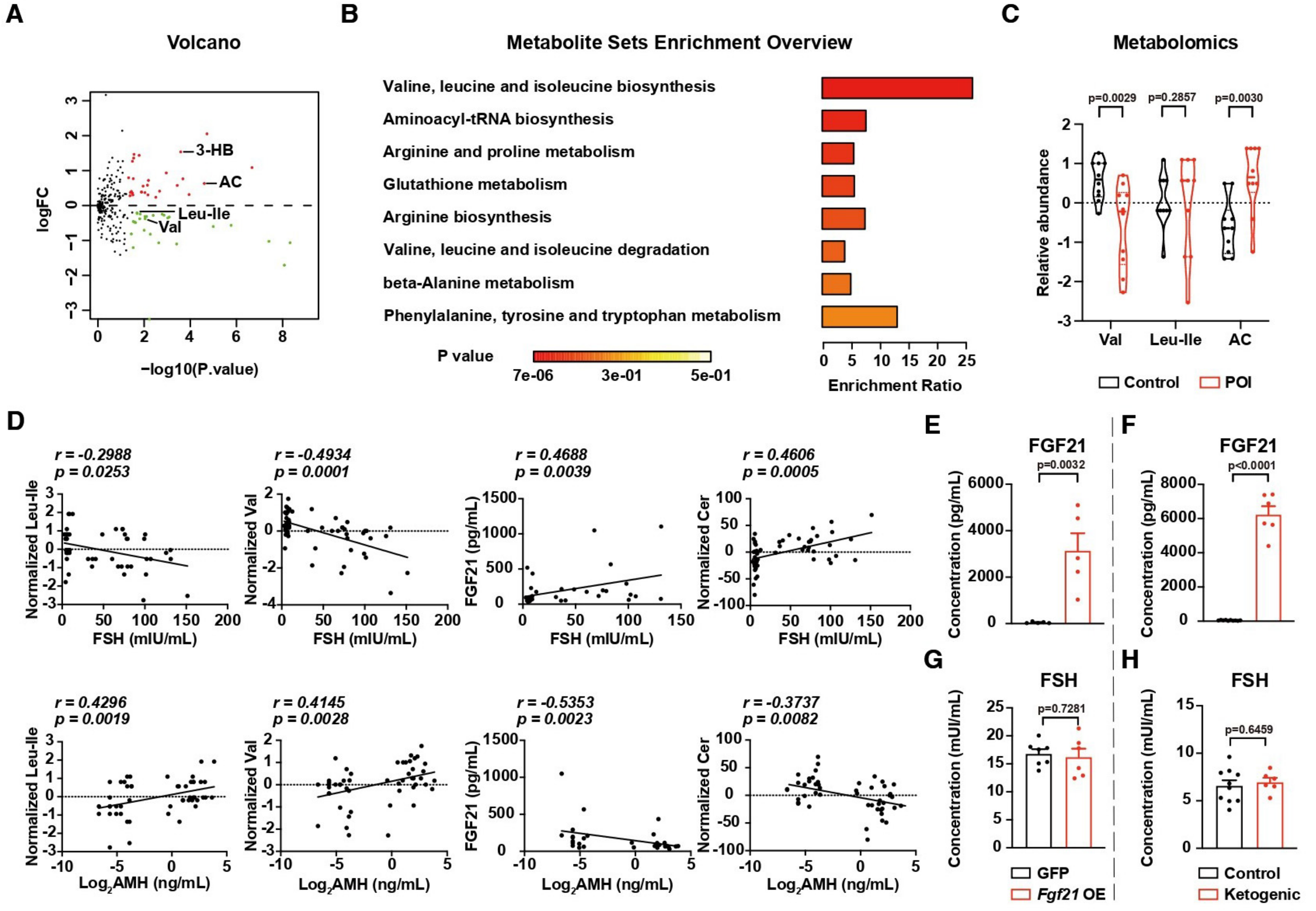

Figure EV1. Metabolic details of POI patients.

A Volcano plot showing the fold-change and *P*-value of metabolites. $N = 18$.

B Enrichment score and *P*-value of metabolites downregulated in POI patients. $N = 18$.

C Relative abundance of valine (Val), acetyl-carnitine (AC) but not leucine-isoleucine (Leu-Ile) in the serum of the Shandong Cohort. $N = 10$; Truncated violin plot, central band stands for median, and dotted lines stand for the upper quartile or the lower quartile of the data.

D The correlations between metabolites/FGF21 and clinical parameters. The *P* and *r* were calculated by the nonparametric Spearman test. $N = 56$.

E, F The concentration of FGF21 in mouse serum. (E) $N = 5$; (F) Control $N = 10$; Ketogenic diet, $N = 6$.

G, H The concentration of FSH in mouse serum. (G) Control, $N = 7$; *Fgf21* OE, $N = 6$; and (H) control, $N = 10$; Ketogenic diet, $N = 6$.

Data information: Error bars stand for SEM. The *P*-value was calculated by a two-tailed *t*-test with 2-way ANOVA correction.
Source data are available online for this figure.

 © 2023 The Authors

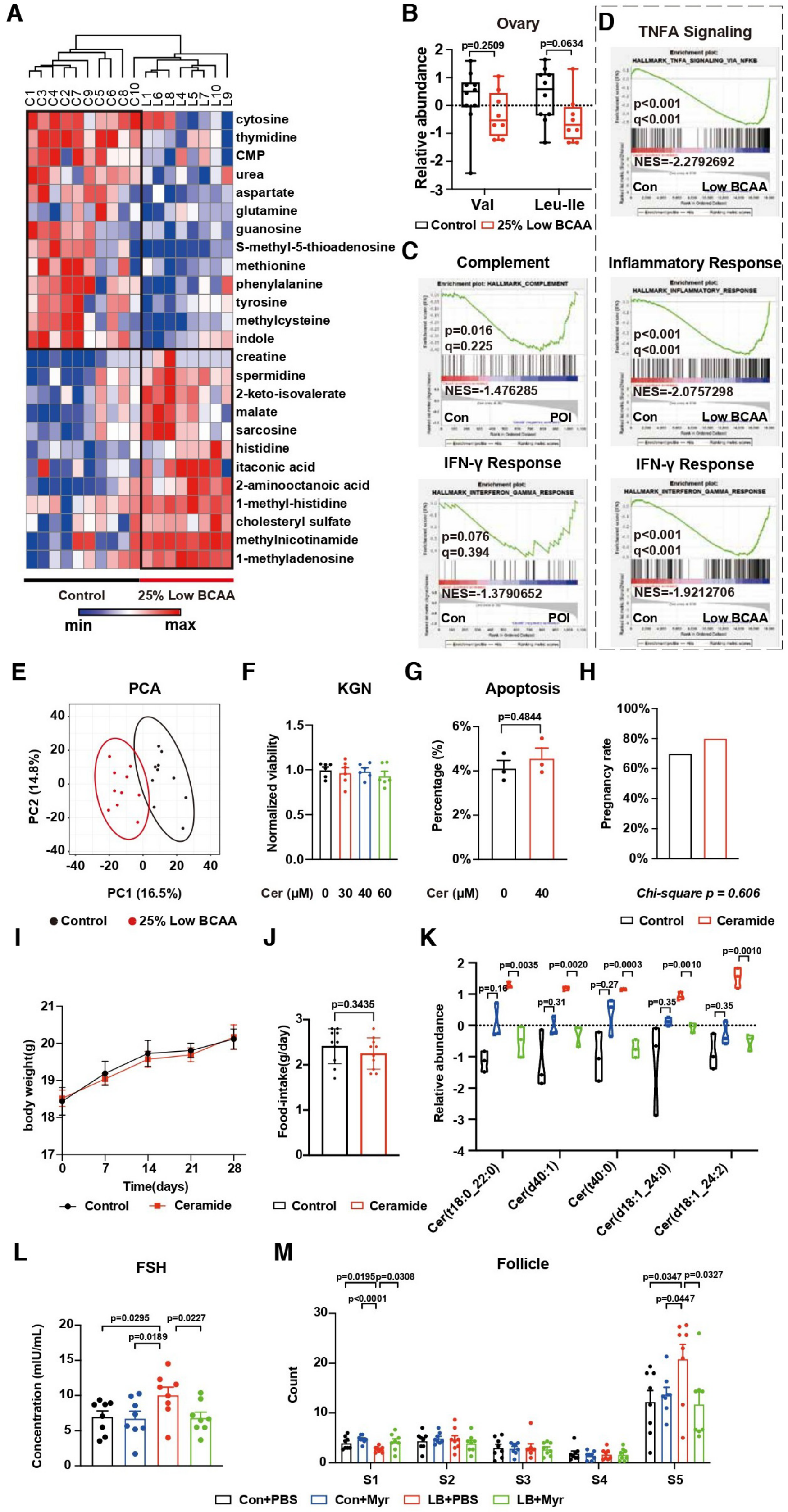

Figure EV2. Lipid metabolism features of POI.

A Heatmap showing the top 25 differentially abundant metabolites in the ovaries of mice on a low BCAA diet. Control, *N* = 10; low BCAA, *N* = 8.

B The relative abundance of BCAAs in the ovaries of mice on a low BCAA diet. Control, *N* = 10; low BCAA, *N* = 8; Boxplot, central band stands for median, boxes stand for 50% of the data, and whiskers stand for min or max of the data.

C GSEA of the proteomics data in the serum of the Fudan Cohort. *N* = 11.

D GSEA of RNA-seq data from the liver in the mice on a low BCAA diet or control diet. *N* = 6.

E The PCA of serum from mice on a low BCAA diet or control diet. *N* = 10.

F The relative cell viability of KGN cells with ceramide treatment. *N* = 6.

G The percentage of Annexin V positive cells of KGN cells with ceramide treatment. *N* = 3.

H–J The pregnancy rate, body weight, and food intake from mice with ceramide treatment. *N* = 10.

K The relative abundance of ceramide in the serum of mice with myriocin treatment. *N* = 3; Truncated violin plot, central band stands for median, and dotted lines stand for the upper quartile or the lower quartile of the data.

L The serum concentration of FSH in mice with ceramide treatment. *N* = 8.

M The changes in follicle count from mice with ceramide treatment. *N* = 8.

Data information: S1, Primordial; S2, Primary; S3, Secondary; S4, Antral; S5, Atretic. Error bars stand for SEM. The *P*-value was calculated by a two-tailed *t*-test with 2-way ANOVA correction.
Source data are available online for this figure.

© 2023 The Authors

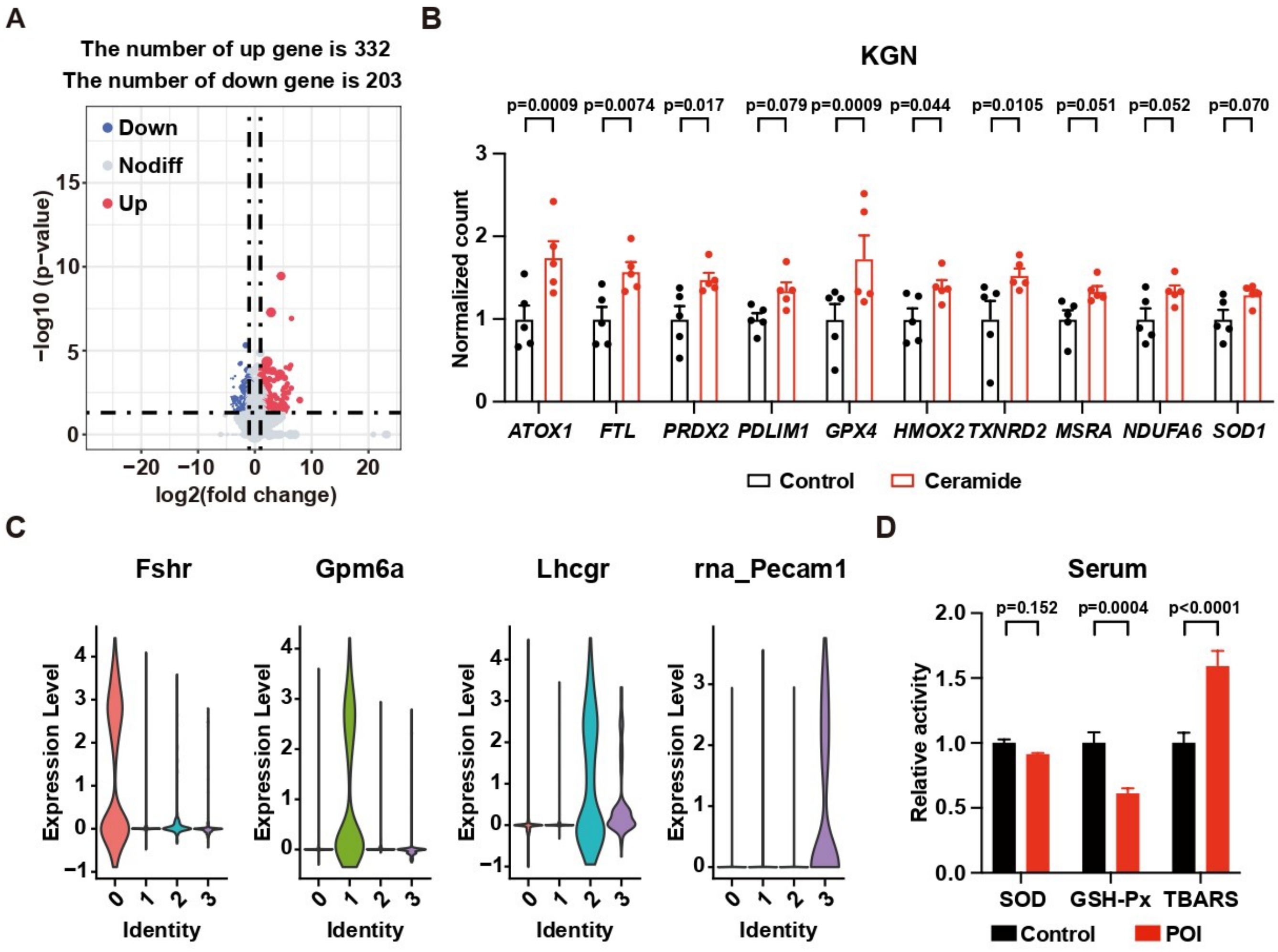

Figure EV3. Low BCAA induces POI via elevation of ROS.

A Genes with two-fold changes and *P*-value < 0.05 are highlighted in the volcano plot. $N = 5$.

B Upregulation of ROS-related genes in KGN cells treated with ceramide. $N = 5$.

C Violin plots showing the expression of classical markers of nonimmune cells in ovaries from sNuc-seq data.

D Relative activity of ROS-related factors in patients. Control, $N = 30$; POI, $N = 60$.

Data information: Error bars stand for SEM. The *P*-value was calculated by a two-tailed *t*-test with 2-way ANOVA correction.
Source data are available online for this figure.

 © 2023 The Authors

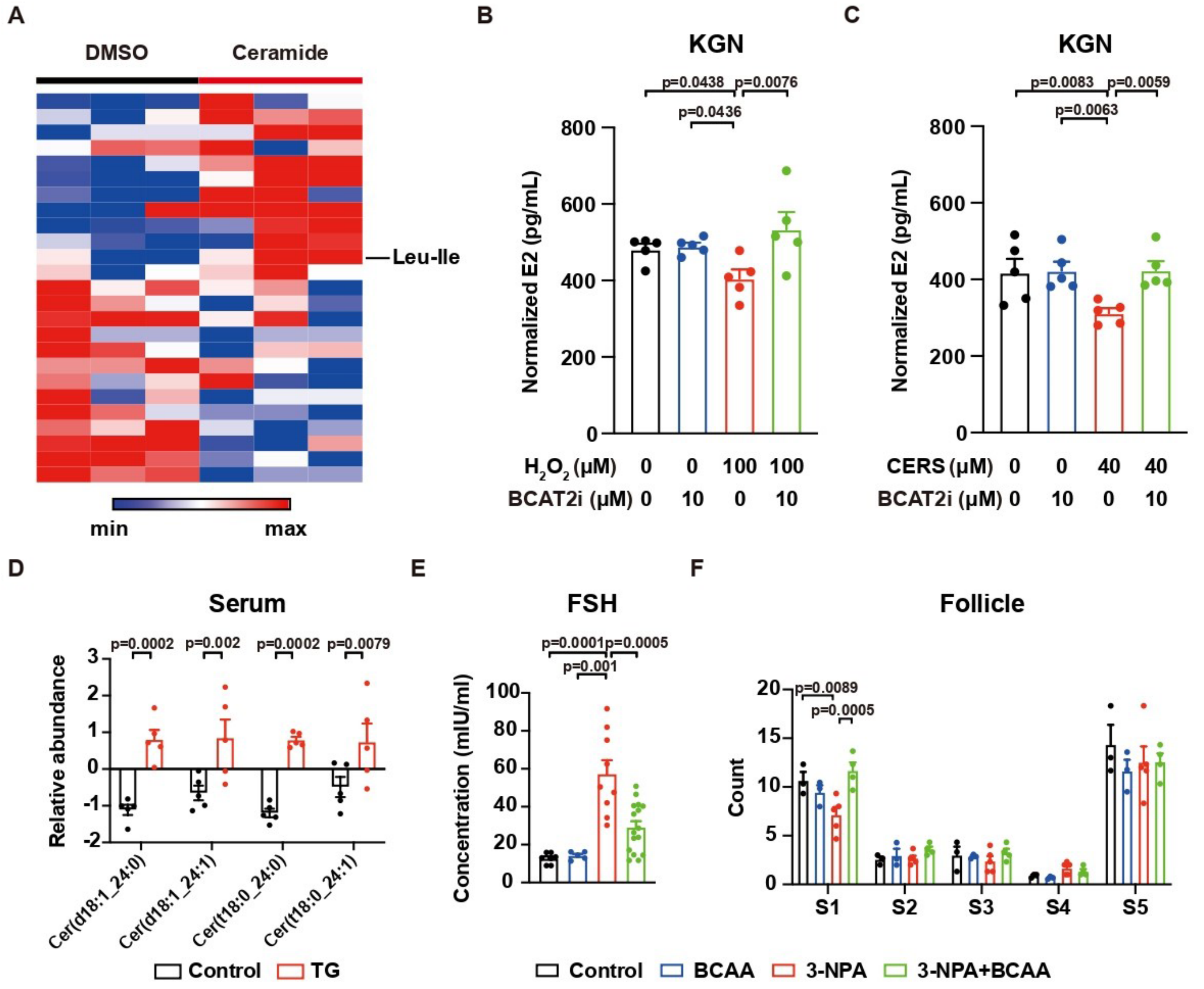

Figure EV4. BCAA supplement protected the granulosa cells from ROS inducer.

A Heatmap showing the relative abundance of metabolites in KGN cells treated with ceramides. *N* = 3.
B The concentration of E2 secreted by KGN cells with H_2O_2 treatment w/o BCAT2 inhibitor. *N* = 5.
C The concentration of E2 secreted by KGN cells with ceramide treatment w/o BCAT2 inhibitor. *N* = 5.
D Elevation of ceramide in the serum of TG-treated mice. *N* = 5.
E BCAA supplement prevented the elevation of FSH in mice with 3-NPA treatment. Control, *N* = 7; BCAA, *N* = 5; 3-NPA, *N* = 9; 3-NPA + BCAA, *N* = 16.
F BCAA supplement rescued the decrease of primordial follicles in mice with 3-NPA treatment. Control, *N* = 3; BCAA, *N* = 3; 3-NPA, *N* = 5; 3-NPA + BCAA, *N* = 4.

Data information: S1, Primordial; S2, Primary; S3, Secondary; S4, Antral; S5, Atretic. Error bars stand for SEM. The *P*-value was calculated by a two-tailed *t*-test with 2-way ANOVA correction.
Source data are available online for this figure.
