# Supplementary material for: BCAA insufficiency leads to premature ovarian insufficiency via ceramide‐induced elevation of ROS
Source: EMBO Mol Med. 2023 Feb 27;15(4):e17450. doi: 10.15252/emmm.202317450 (PMC10086587; doi:10.15252/emmm.202317450)
Supplement: Supplementary file 17 — PDF+ [file EMMM-15-e17450-s005.pdf]

Article

SOURCE DATA | TRANSPARENT PROCESS | OPEN ACCESS

EMBO Molecular Medicine

BCAA insufficiency leads to premature ovarian insufficiency via ceramide-induced elevation of ROS

Xiao Guo[1,†], Yuemeng Zhu[1,2,†], Lu Guo[1,2,†], Yiwen Qi[1,2,3,†], Xiaocheng Liu[1,2], Jinhui Wang[1], Jiangtao Zhang[4,5], Linlin Cui[4,5], Yueyang Shi[6], Qichu Wang[1], Cenxi Liu[1], Guangxing Lu[1], Yilian Liu[1], Tao Li[6], Shangyu Hong[1], Yingying Qin[3,4], Xuelian Xiong[1], Hao Wu[1], Lin Huang[1], He Huang[1,*], Chao Gu[1,2,**], Bin Li[1,2,***] & Jin Li[1,****,‡]

Abstract

Premature ovarian insufficiency (POI) is a disease featured by early menopause before 40 years of age, accompanied by an elevation of follicle-stimulating hormone. Though POI affects many aspects of women's health, its major causes remain unknown. Many clinical studies have shown that POI patients are generally underweight, indicating a potential correlation between POI and metabolic disorders. To understand the pathogenesis of POI, we performed metabolomics analysis on serum and identified branch-chain amino acid (BCAA) insufficiency-related metabolic disorders in two independent cohorts from two clinics. A low BCAA diet phenotypically reproduced the metabolic, endocrine, ovarian, and reproductive changes of POI in young C57BL/6J mice. A mechanism study revealed that the BCAA insufficiency-induced POI is associated with abnormal activation of the ceramide-reactive oxygen species (ROS) axis and consequent impairment of ovarian granulosa cell function. Significantly, the dietary supplement of BCAA prevented the development of ROS-induced POI in female mice. The results of this pathogenic study will lead to the development of specific therapies for POI.

Keywords ceramide; infertility; low BCAA diet; premature ovarian insufficiency; ROS
Subject Categories Metabolism; Urogenital System
DOI 10.15252/emmm.202317450 | Received 18 January 2023 | Revised 31 January 2023 | Accepted 31 January 2023 | Published online 27 February 2023
EMBO Mol Med (2023) 15: e17450

Introduction

Premature ovarian insufficiency (POI) is a disease featured by early menopause before 40 years of age with follicle-stimulating hormone (FSH) > 25 U/l. The ovaries of POI patients usually present with fewer primordial follicles and more atretic follicles, indicating accelerated follicular atresia/destruction or defects in supporting, recruitment, and maturation of primordial/growing follicles (Ishizuka, 2021; Lambrinoudaki *et al*, 2021; McGlacken-Byrne & Conway, 2022). Consequently, women with POI suffer from subfertility and are susceptible to estrogen deficiency-related aging symptoms in the bone, cardiovascular system, and central nervous system (Tsiligiannis *et al*, 2019; Samad *et al*, 2020; Stevenson *et al*, 2021). Hormone replacement therapy (HRT) can certainly alleviate these symptoms (Luisi *et al*, 2015; Webber *et al*, 2017; Armeni *et al*, 2021), though the therapies to prevent or cure POI itself are still absent.

A few POI patients have presented with a relevant family medical history (Vegetti *et al*, 1998; Bachelot *et al*, 2009; Tucker *et al*, 2016). Genetic studies revealed that their genomes contain mutations within genes that were critical for ovarian function. However, a portion of POI patients has no relevant family medical history (Chapman *et al*, 2015), and the mechanism of these sporadic cases of POI remains unclear. In comparison to the large body of observational studies, research systemically investigating the cause of POI is very limited. A better understanding of its pathogenesis is important to develop specific therapies, other than HRT, to prevent or cure POI.

1 Obstetrics and Gynecology Hospital, State Key Laboratory of Genetic Engineering, School of Life Sciences, Zhongshan Hospital and Institute of Metabolism and Integrative Biology, Fudan University, Shanghai, China
2 Shanghai Key Laboratory of Female Reproductive Endocrine Related Diseases, Shanghai, China
3 Shanghai First Maternity and Infant Hospital, Shanghai, China
4 Center for Reproductive Medicine, Cheeloo College of Medicine, Shandong University, Jinan, China
5 Key Laboratory of Reproductive Endocrinology of Ministry of Education, National Research Center for Assisted Reproductive Technology and Reproductive Genetics, Jinan, China
6 Shandong Provincial Hospital, Cheeloo College of Medicine, Shandong University, Jinan, China
*Corresponding author. Tel: +86 021 31242078; E-mail: he_huang@fudan.edu.cn
**Corresponding author. Tel: +86 021 33189900; E-mail: drchaogu@126.com
***Corresponding author. Tel: +86 021 33189900; E-mail: binli@fudan.edu.cn
****Corresponding author. Tel: +86 021 31246513; E-mail: li_jin_lifescience@fudan.edu.cn
[†]These authors contributed equally to this work
[‡]This author contributed equally to this work as senior author

© 2023 The Authors. Published under the terms of the CC BY 4.0 license

The recent white paper on POI from the International Menopause Society (Panay *et al*, 2020) and results from several clinical studies (Michalakis & Coppack, 2012; Szegda *et al*, 2017) showed that being underweight was a key feature of POI patients, which suggests a correlation between POI and metabolic disorders. Since most POI patients in the clinic have been exposed to some kind of HRT, which dramatically affect their metabolism, it is difficult to collect proper samples for comprehensive analysis. In this study, we investigated the metabolic changes of POI patients who had never been exposed to HRT by liquid chromatography–mass spectrometry (LC–MS)-based metabolomics. We found low serum branch chain amino acid (BCAA) levels in these patients, which was validated in an independent cohort collected in a different center. With multiple models, we validated that BCAA abundance regulates ovarian function and fertility via the effects of the ceramide-reactive oxygen species (ROS) axis on ovarian granulosa cells. Additionally, dietary supplementation with BCAA protects ovaries from ROS-induced POI in mice.

Results

Metabolic disorders in POI patients

A cohort of 18 POI patients without relevant family history and matched healthy donors was established (Fudan Cohort). None of the patients had ever received HRT previously. The general clinical information is presented in Fig 1A. We then profiled the metabolic changes of these patients and healthy controls by performing LC–MS-based targeted metabolomics of the serum (Dataset EV1). Overall, POI patients showed different metabolic features in principle component analysis (PCA) (Fig 1B). The distinct separation of the top-75 differentially detected metabolites was presented on the heatmap (Fig 1C). The significantly changed metabolites are highlighted in the volcano plot in Fig EV1A.

Unbiased functional enrichment analysis found the downregulated metabolites were enriched in amino acid metabolism (Fig EV1B). Specifically, we observed decreases in the level of BCAAs, including leucine-isoleucine and valine (Fig 1D). It is known that BCAA deficiency may induce an enhancement of ketogenesis. Consistently, the ketogenesis-related metabolites 3-hydroxybutyrate and acetyl-carnitine were also upregulated in POI patients (Fig 1E). Importantly, similar changes in valine and acetyl-carnitine were observed in an independent cohort (Shandong Cohort) with 10 POI patients without exposure to HRT and matched healthy donors from the Center for Reproductive Medicine in Shandong University (Fig EV1C, Table EV1, and Dataset EV2). We also found elevated ketogenesis-related secreted protein FGF21 (Fig 1F) but not GDF15 (Fig 1G) in the Fudan Cohort. In addition, significant correlations between the serum concentration of the metabolites/FGF21 and FSH/AMH were observed in Fudan Cohort (Fig EV1D).

The effects of ketogenesis or elevated FGF21 on ovarian function are rather controversial (Owen *et al*, 2013; Singhal *et al*, 2016; Zhuo *et al*, 2019), but the correlation between FGF21 or ketogenesis and onset of POI has not been explored. We found that neither *Fgf21* overexpression (*Fgf21* OE) nor 2 months of a ketogenic diet fed to young female mice induced significant changes in serum FSH level (Fig EV1E–H), suggesting neither increased FGF21 nor elevated ketogenesis directly induced POI.

BCAA insufficiency leads to POI-like metabolic changes in young female mice

We then tested the effects of BCAA insufficiency by feeding mice with a low BCAA diet. We first confirmed the amino acid abundance (Appendix Fig S1A) and energy content (Appendix Fig S1B) in the diet. The detailed recipe of the low BCAA diet is presented in Appendix Fig S1C. It has been shown that a low BCAA diet can lead to dramatic changes in metabolism, including an increase in energy expenditure, enhancement of ketogenesis, upregulation of *Fgf21*, and a decrease in body weight in mice (Newgard *et al*, 2009; Lotta *et al*, 2016; Cummings *et al*, 2018; Karusheva *et al*, 2019; Zhou *et al*, 2019; Richardson *et al*, 2021; Yu *et al*, 2021). However, its effects on young lean female mice, which are commonly used for studying POI, have not been reported.

So, we benchmarked its overall effects on metabolism at first. A low BCAA (25%) diet over the long-term (3 months), but not short-term (1.5 months), led to a decrease in body weight (Appendix Fig S2A and B). The low BCAA diet did decrease BCAA in the serum (Appendix Fig S2C), increase energy expenditure (Appendix Fig S2D), change the abundance of metabolites related to ketogenesis (Appendix Fig S2E), upregulate the level of *Fgf21* in the liver (Appendix Fig S3F), and increase FGF21 in serum (Appendix Fig S2G). These results indicate that low BCAA diet-induced POI-like changes in the metabolism of young lean female mice.

BCAA insufficiency leads to POI

The effects of BCAA insufficiency on ovarian function and fertility have not been explored previously. Intriguingly, we found that young female mice fed by a low BCAA diet for 1.5 months developed POI-like phenotypes including upregulation of FSH (Fig 2A), downregulation of primordial follicles, and upregulation of atretic follicles (Fig 2B). Notably, the elevation of FSH was also observed with the diet from a different batch (Fig 2C) and from a different vendor (Fig 2D). The features of the diet from a different vendor were presented in Appendix Fig S1D.

Although feeding with the low BCAA diet for 1.5 months did not affect the level of estraone (E2) and anti-Müllerian hormone (AMH) (Fig 2E and F), prolonged feeding (3 months) led to a significant decrease in E2 (Fig 2G) and AMH (Fig 2H). In addition, the lower pregnancy rates (Fig 2I) and the absence of pups (Fig 2J) were found in mice on the low BCAA diet. These data suggest BCAA insufficiencies, but not the elevation of ketogenesis or FGF21 induces POI in mice.

Although several studies used a low BCAA diet (under 25%) for metabolic research previously (Purpera *et al*, 2012; Cummings *et al*, 2018), many studies also used a milder diet with 33% BCAA or even higher (Tournissac *et al*, 2018; Richardson *et al*, 2021; Yu *et al*, 2021). We discovered the 50% low BCAA diet also led to remarkable upregulation of FSH (Appendix Fig S3A), downregulation of primordial follicles, and upregulation of atretic follicles (Appendix Fig S3B). We also tested the effects of a 25% low BCAA diet on 7-month-old female mice. We observed a consistent elevation of serum FSH levels (Appendix Fig S3C), together with the downregulation of primordial follicles and upregulation of atretic follicles (Appendix Fig S3D). These results indicate BCAA insufficiency can significantly induce POI.

 © 2023 The Authors

A

Patient information for this study.

		Control	POI	P value
**Group size**		18	18	
**Age (years)**		28.20±2.23	32.17±4.91	>0.05
**BMI (kg/m²)**		20.54±2.14	22.09±3.09	>0.05
**Menstruation**		regular	irregular	<0.0001
**FSH (mIU/mL)**		6.50±1.88	73.04±33.49	<0.0001
**AMH (ng/mL)**	NO	5	2	
	YES	6.46±3.37	0.14±0.28	<0.0001
**E2 (pg/mL)**		201.33±339.12	67.03±135.06	0.1433
**Pelvic Surgery**		0	0	>0.05
**Metabolic Disease**	Diabetes	0	0	>0.05
	PCOS	0	0	>0.05
	Fatty liver	2	0	>0.05
	hyperthyroidism	0	0	>0.05
	hypothyroidism	0	0	>0.05
	Hypertension	0	0	>0.05
	gout	0	0	>0.05
**ALT (U/L)**		13.39±5.42	19.35±9.33	0.0350

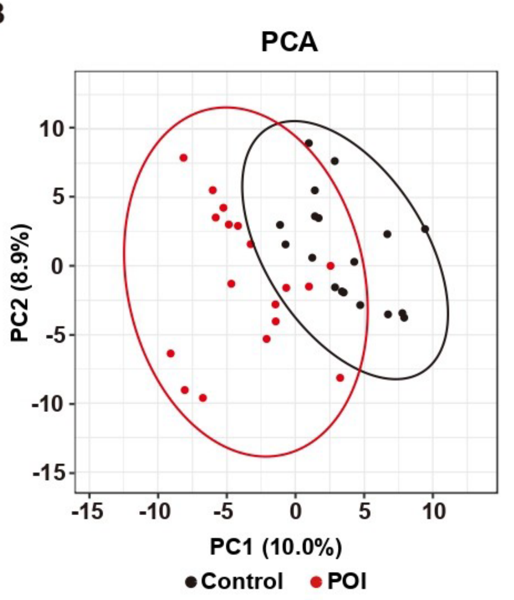

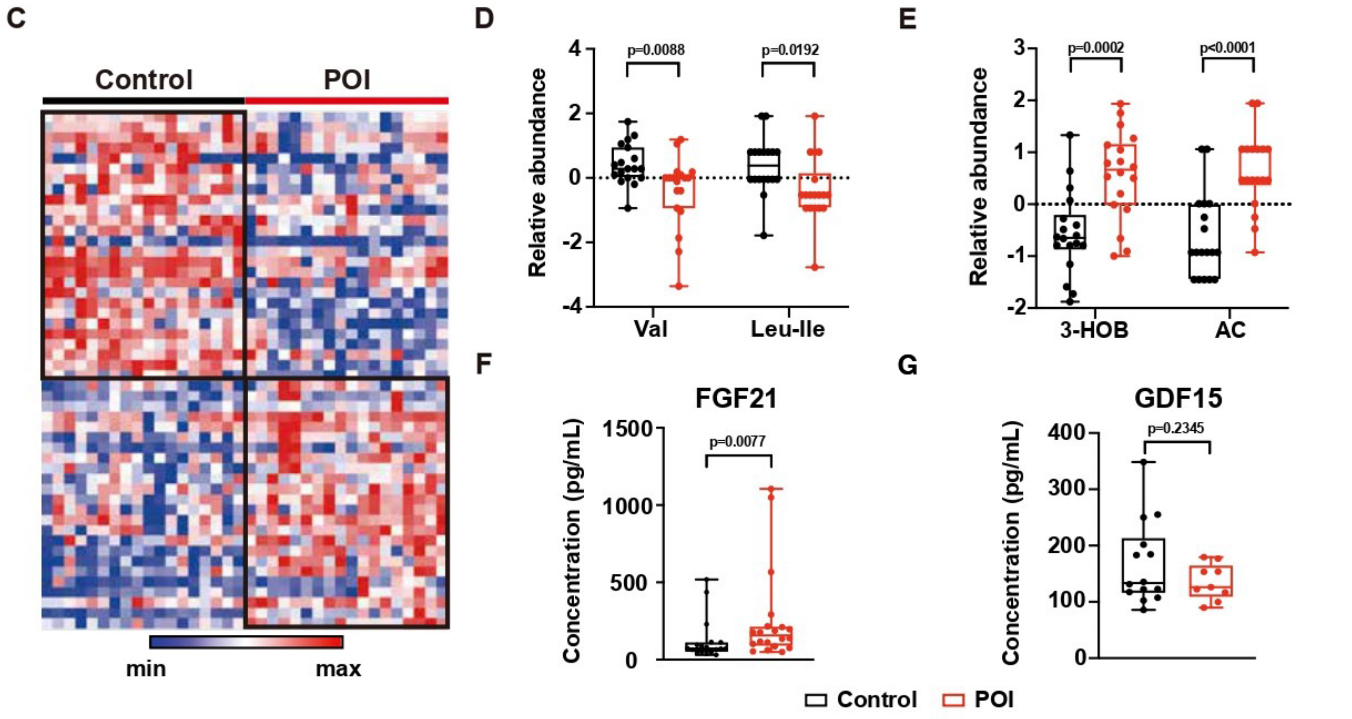

Figure 1. Metabolic disorders in POI patients.

A Clinical information of the cohort.

B PCA of serum metabolomics. Circles indicate 95% confidence intervals. $N = 18$.

C Heatmap showing the relative abundance of the top 75 differential expressed metabolites. $N = 18$.

D The relative abundance of valine (Val) and leucine-isoleucine (Leu-Ile). $N = 18$; Boxplot, central band stands for median, boxes stand for 50% of the data, and whiskers stand for min or max of the data.

E The relative abundance of 3-hydroxybutyrate (3-HOB) and acetylcarnitine (AC). $N = 18$; Boxplot, central band stands for median, boxes stand for 50% of the data, and whiskers stand for min or max of the data.

F The concentration of FGF21 in serum. Control, $N = 18$; POI, $N = 20$; Boxplot, central band stands for median, boxes stand for 50% of the data, whiskers stand for min or max of the data.

G The concentration of GDF15 in serum. Control, $N = 14$; POI, $N = 9$; Boxplot, central band stands for median, boxes stand for 50% of the data, and whiskers stand for min or max of the data.

Data information: Error bars stand for SEM of biological repeats. The *P*-value was calculated by a two-tailed *t*-test with 2-way ANOVA correction.
Source data are available online for this figure.

© 2023 The Authors

Elevation of ceramide in POI impairs ovarian granulosa cell function

We next explored how BCAA insufficiencies lead to POI. We found the BCAAs were absent from the top 25 differentially changed metabolites in the ovaries of mice on a low BCAA diet (Fig EV2A), though there was an insignificant difference (Fig EV2B). Despite the systemic BCAA insufficiency, these data indicated that BCAA insufficiency may not be the direct inducer of POI. We further explored the mechanism of BCAA insufficiency-induced POI. It has been

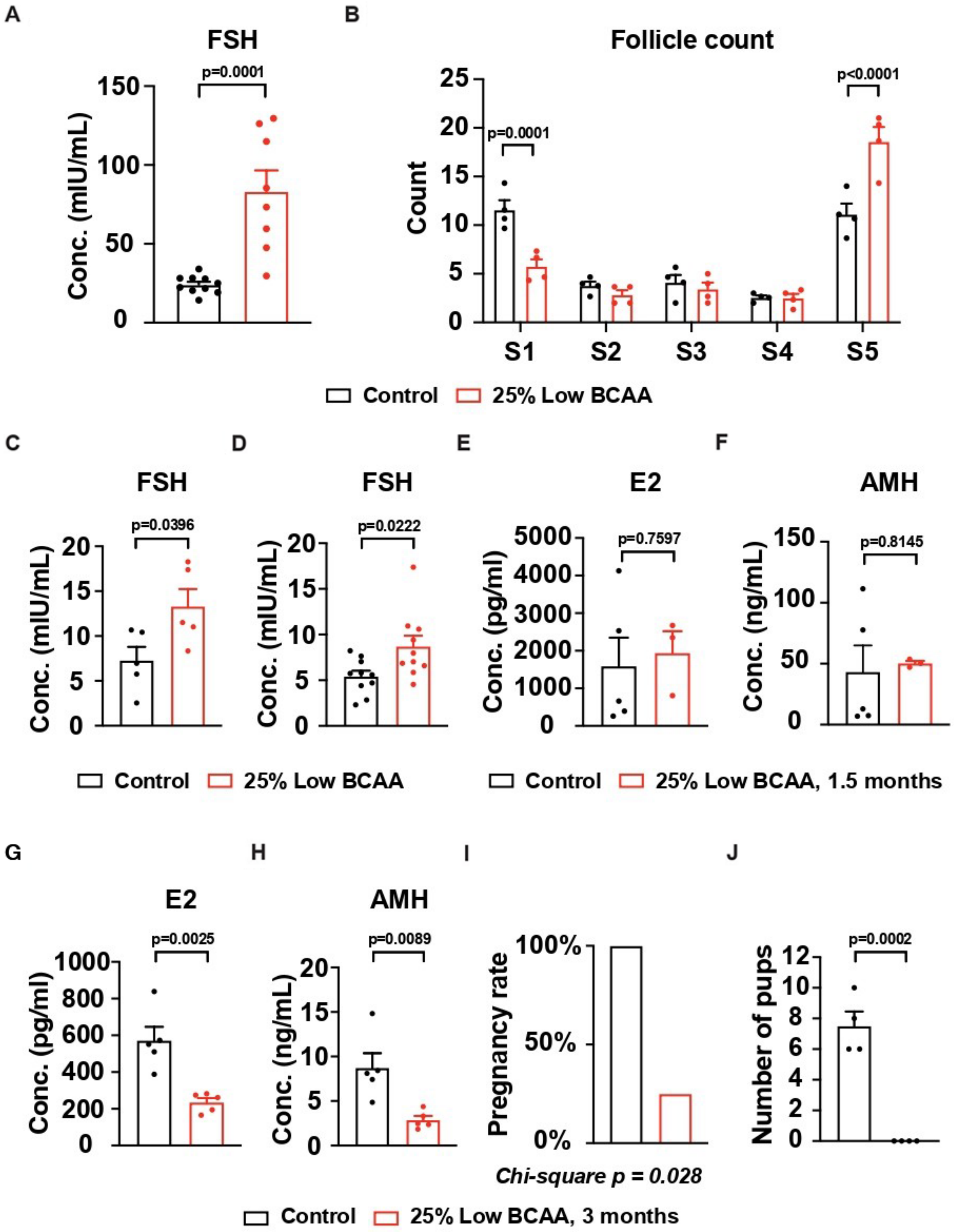

Figure 2.

© 2023 The Authors

Figure 2. Low BCAA diet induces POI-like phenotypes in young female mice.

A, B The serum concentration of FSH and the changes of follicles in mice on a low BCAA diet for 1.5 months. (A) Control, $N = 10$; low BCAA, $N = 8$; (B) Control, $N = 4$; low BCAA, $N = 4$.
C The serum concentration of FSH in mice on a different batch of the low BCAA diet. $N = 5$.
D The serum concentration of FSH in mice on the low BCAA diet from Research Diet. $N = 10$.
E, F The serum concentration of AMH and E2 in mice on the low BCAA diet for 1.5 months. Control, $N = 5$; low BCAA, $N = 3$.
G, H The serum concentration of AMH and E2 in mice on the low BCAA diet for 3 months. $N = 5$.
I, J The pregnancy rate and number of pups per pregnancy on the low BCAA diet for 3 months. $N = 4$.

Data information: S1, Primordial; S2, Primary; S3, Secondary; S4, Antral; S5, Atretic. Error bars stand for SEM of biological repeats. The *P*-value was calculated by two-tailed *t*-test with 2-way ANOVA correction.
Source data are available online for this figure.

proposed that dietary BCAA is correlated with inflammation (Papathanassiu *et al*, 2017; Zhenyukh *et al*, 2017; Cosentino *et al*, 2021). We observed the elevation of proteins enriched in inflammation-related gene sets in the proteomics analysis of POI patients' serum by Gene Set Enrichment Analysis (GSEA) (Fig EV2C and Dataset EV3). GSEA also revealed the upregulation of genes enriched in inflammation-related gene sets of the liver from mice on a low BCAA diet (Fig EV2D and Dataset EV4).

In the context of metabolic research, it has been reported that ceramide synthase activity is activated by exposure to pro-inflammatory cytokines (Hernandez-Corbacho *et al*, 2015; Ottenlinger *et al*, 2016). Interestingly, we found the upregulation of various kinds of ceramide in POI patients' serum with LC–MS-based untargeted lipidomics (Fig 3A and Dataset EV5). Similar changes in ceramide were also identified in the Shandong Cohort (Fig 3B and Dataset EV6) and mouse serum (Figs 3C and EV2E, and Dataset EV7). Importantly, we also discovered increased ceramides in the ovaries of mice fed on a low BCAA diet (Fig 3D and Dataset EV8).

We then tested the effects of ceramide on ovarian granulosa cells, which are the major cell type responsible for POI. Ceramide treatment did not affect cell viability (Fig EV2F) and the proportion of apoptosis marker Annexin V positive cells (Fig EV2G), but dramatically attenuated the capability of E2 secretion (Fig 3E) in KGN cells of the human granulosa cell line. Notably, ceramide treatment *in vivo* led to elevated serum concentrations of FSH (Fig 3F), no obvious changes in pregnancy rate, body weight, and food intake (Fig EV2H–J), a reduction of pups (Fig 3G), downregulation of primordial follicles and upregulation of atretic follicles (Fig 3H). In contrast, the application of myriocin (Myr), a known inhibitor of ceramide (Lin *et al*, 2018; Yang *et al*, 2019; Woo *et al*, 2020), rescued the serum level of ceramide, the elevation of FSH and changes of follicles (Fig EV2K–M) induced by low BCAA diet. Thus, we conclude that BCAA insufficiency-induced ceramide elevation is one of the pathogenic factors for POI.

Ceramide impairs granulosa cell function via enhancement of ROS

We further investigated the mechanism of ceramide-induced POI. RNA-seq analysis identified 535 genes with significant changes in KGN cells induced by ceramide (Fig EV3A and Dataset EV9). GSEA revealed ceramide treatment upregulated genes related to ROS in KGN cells (Figs 4A and EV3B). Since the ovary is composed of several cell types, the RNA-seq analysis of whole ovaries may not be able to precisely describe the changes in granulosa cells. To validate the changes of ROS in granulosa cells *in vivo*, single nuclei RNA-seq (sNuc-seq) was performed on the ovaries of mice fed by either control or a low BCAA diet. The analysis focusing on nonimmune cells identified granulosa cells (cluster 0), mesothelial cells (cluster 1), thecal cells (cluster 2), and endothelial cells (cluster 3) from the ovaries (Fig 4B). The expression of classic markers for each cell type was presented in Fig EV3C. The low BCAA diet induced the elevation of multiple ROS-related genes in granulosa cells (Fig 4C), indicating upregulation of ROS *in vivo*.

The elevation of ROS in POI patients was also identified by measuring the level of ROS indicators in serum (Fig EV3D and Dataset EV10). Consistently, E2 production was reduced by ROS inducer H_2O_2 in KGN cells (Fig 4D). Metabolomics analysis revealed ceramide treatment disturbed the metabolism of glutathione (GSH) and glutathione disulfide (GSSG) (Fig 4E and F, and Dataset EV11).

Figure 3. Elevation of ceramide impaired granulosa cell function.

A, B Elevation of serum ceramide in POI patients from the Fudan Cohort or the Shandong Cohort. Left, the relative abundance of total ceramide; right, the relative abundance of ceramide with specific acyl chain. (A) $N = 18$; (B) $N = 10$; Truncated violin plot, central band stands for median, and dotted lines stand for the upper quartile or the lower quartile of the data.
C Heatmap showing the top 75 changed lipids in the serum of mice on a low BCAA diet. $N = 10$.
D Elevation of ceramide in the ovaries of mice on a low BCAA diet. $N = 10$; Truncated violin plot, central band stands for median, and dotted lines stand for the upper quartile or the lower quartile of the data.
E Decreases of E2 secretions from KGN cells treated with ceramide. $N = 3$.
F The serum concentration of FSH in mice with ceramide treatment. $N = 7$.
G Number of pups from mice with ceramide treatment. Control, $N = 4$; ceramide treatment, $N = 5$.
H The changes in follicle count from mice with ceramide treatment. $N = 7$.

Data information: S1, Primordial; S2, Primary; S3, Secondary; S4, Antral; S5, Atretic. Error bars stand for SEM of biological repeats. The *P*-value was calculated by a two-tailed *t*-test with 2-way ANOVA correction.
Source data are available online for this figure.

© 2023 The Authors

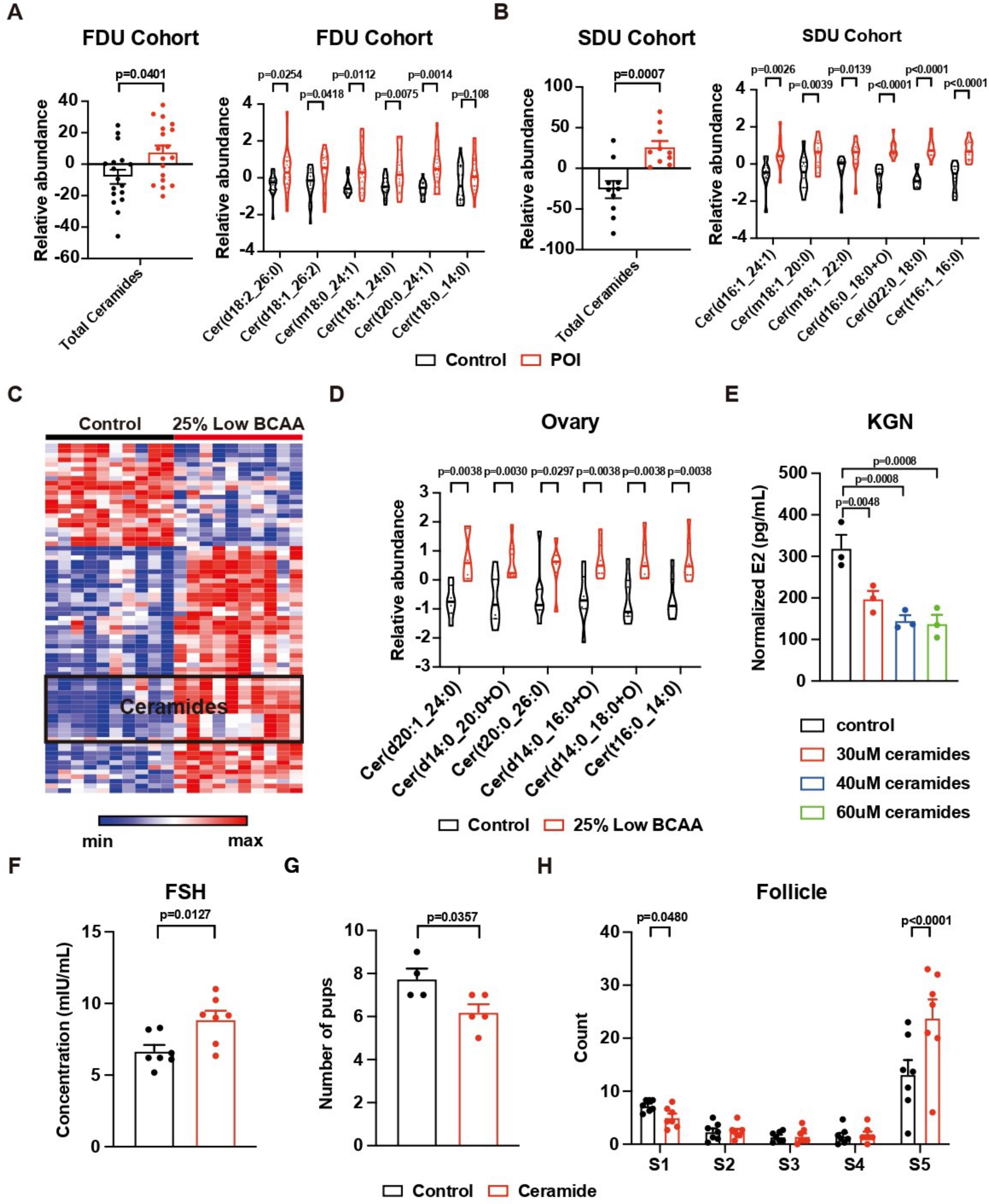

Figure 3.

 © 2023 The Authors

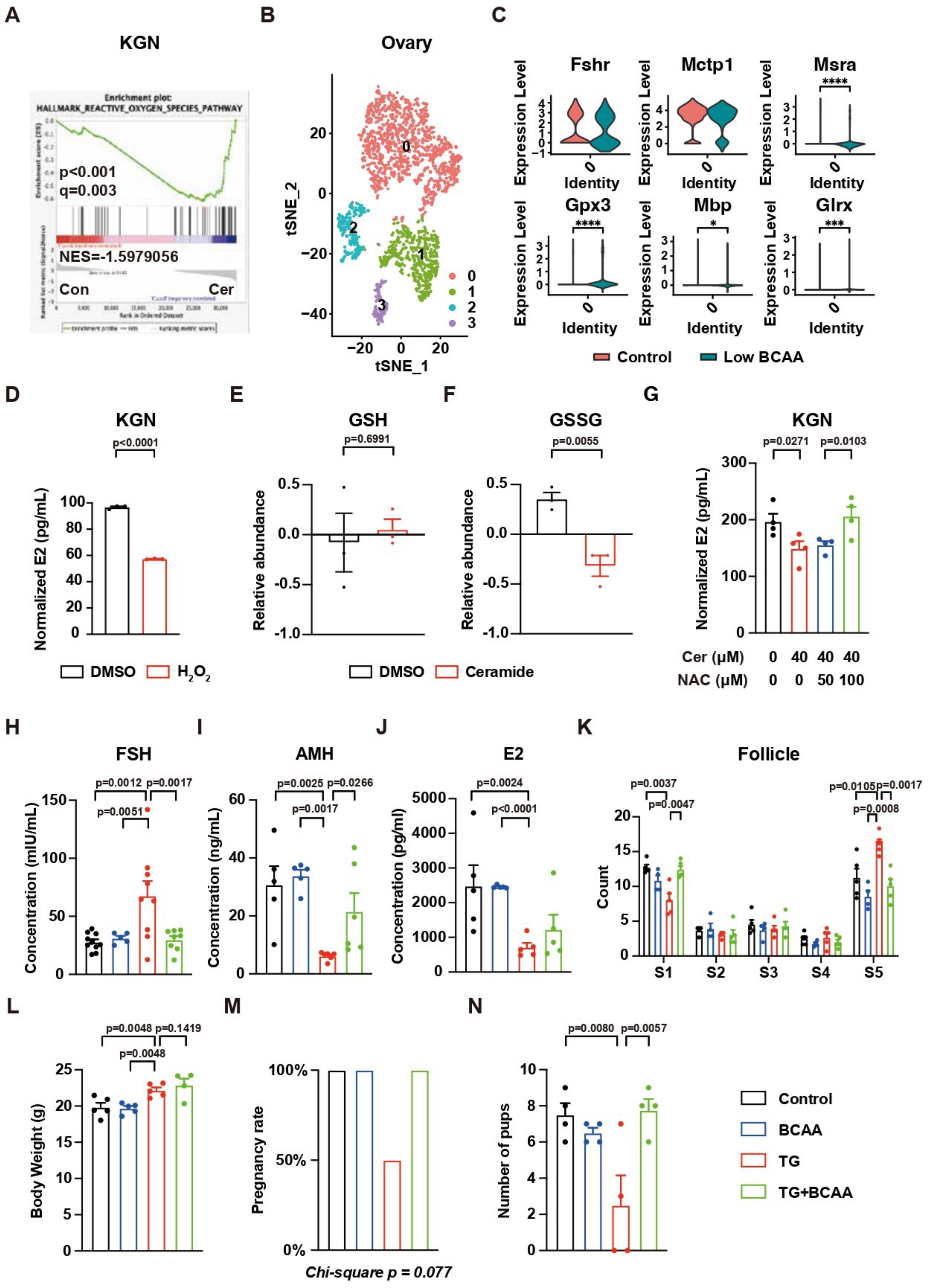

A
KGN
p<0.001
q=0.003
NES=-1.5979056
Con
Cer
B
Ovary
tSNE_2
tSNE_1
C
Fshr
Mctp1
Msra
Gpx3
Mbp
Glrx
Expression Level
Identity
Control
Low BCAA
D
KGN
p<0.0001
Normalized E2 (pg/mL)
DMSO
H2O2
E
GSH
p=0.6991
Relative abundance
F
GSSG
p=0.0055
DMSO
Ceramide
G
KGN
p=0.0271
p=0.0103
Normalized E2 (pg/mL)
Cer (μM)
NAC (μM)
H
FSH
Concentration (mIU/mL)
I
AMH
Concentration (ng/mL)
J
E2
Concentration (pg/ml)
K
Follicle
Count
L
Body Weight (g)
M
Pregnancy rate
Chi-square p = 0.077
N
Number of pups
Control
BCAA
TG
TG+BCAA

Figure 4.

◀ **Figure 4. BCAA-induced POI via ceramide-ROS axis.**

A GSEA results of RNA-seq data. $N = 5$.
B sNuc-seq data of ovarian nonimmune cells. Granulosa cells (cluster 0), mesothelial cells (cluster 1), thecal cells (cluster 2), and endothelial cells (cluster 3).
C, D (C) Relative expression of genes related to ROS in granulosa cells and (D) the concentration of E2 secreted by KGN cells with H_2O_2 treatment. $N = 3$.
E, F Relative abundance of GSH and GSSG. $N = 3$.
G The concentration of E2 secreted by KGN cells with ceramide treatment w/o NAC. $N = 4$.
H–J The serum concentration of FSH, AMH, and E2. (H) Control, $N = 10$; BCAA, $N = 5$; TG = 9; TG + BCAA = 8; (I) Control, $N = 5$; BCAA, $N = 5$; TG = 6; TG + BCAA = 6; (J) $N = 5$.
K The changes in follicle count. Control, $N = 5$; BCAA, $N = 4$; TG = 5; TG + BCAA = 5.
L Body weight. Control, $N = 5$; BCAA, $N = 5$; TG = 5; TG + BCAA = 4.
M Pregnancy rate. $N = 4$.
N Number of pups from mice with TG or TG + BCAA supplement treatment. $N = 4$.

Data information: Error bars stand for SEM of biological repeats. The *P*-value was calculated by a two-tailed *t*-test with 2-way ANOVA correction.
Source data are available online for this figure.

Supplementing with the GSH precursor N-acetylcysteine (NAC) prevented the downregulation of E2 production upon ceramide treatment (Fig 4G). These results from cellular experiments suggested a direct effect of ROS on the development of POI.

BCAA supplement protects ovaries from ROS-induced POI

The protective effects of NAC on KGN cells inspired us to validate its effects *in vivo*. However, we observed that NAC treatment led to a rapid decrease in body weight and a decrease in activity in young lean female mice, which limited its potential for clinical application. Intriguingly, the metabolomics analysis also showed increases in BCAA in KGN cells treated with ceramide (Fig EV4A). We hypothesize that the increase of BCAA in KGN cells is a protective response against ceramide-induced elevation of ROS. Similar phenotypes have been observed in the skeletal muscle (He & Zhang, 2022; Yao *et al*, 2022). Interestingly, prevention of BCAA catabolism by Branched Chain Amino Acid Transaminase 2 inhibitor (BCAT2i) rescued the decreases of E2 production induced by either H_2O_2 or ceramide (Fig EV4B–C).

We further tested the interaction between ROS and BCAA in the onset of POI *in vivo*. We found the elevation of serum ceramide in female mice treated with a ROS-inducer tripterygium glycosides (TG) (Fig EV4D), which was a similar phenotype to BCAA deficient mice. Thus, we tested the effects of dietary supplementation of BCAA on ROS-inducer TG or another ROS inducer 3-nitropropionic acid (3-NPA) treated mice. The supplement of BCAA via drinking water prevented TG or 3-NPA-induced POI phenotypes, including changes in hormones (Figs 4H–J and EV4E) as well as primordial follicles and atretic follicles amount (Figs 4K and EV4F). The BCAA supplement did not dramatically affect body weight (Fig 4L). Importantly, the fertility was preserved by the BCAA supplement, as a trend of elevation in the pregnancy rate (Fig 4M) and a significant increase in the number of pups (Fig 4N). Despite its potential side effects, our data suggest that BCAA supplements can protect the ovaries from ROS-induced POI.

Discussion

Understanding the pathogenesis of POI is the key to developing therapies for its prevention and treatment. Our study revealed that BCAA insufficiency-induced metabolic disorders contributed to the onset of POI. It is well known that reproductive function is tightly regulated by metabolic homeostasis. For example, excessive loss of body weight may induce amenorrhea, which may be corrected by a lifestyle change (Huhmann, 2020; Strock *et al*, 2020; Riva *et al*, 2021). However, to the best of our knowledge, POI has not been linked to any form of malnutrition in epidemiological studies. The reasons for BCAA insufficiency in POI patients, possibly correlated to genetic variants (Xu *et al*, 2013; Boulet *et al*, 2015) or gut microbiota changes (Pedersen *et al*, 2016), are still unclear and need to be investigated.

In the past decades, many studies have revealed the nutrient composition of the diet is important for homeostasis. Specifically, it has been reported the amino acid sensing protein mTOR is an important factor in the development of POI (Rehnitz *et al*, 2022; Xu *et al*, 2022). These results suggested that amino acid metabolism might have significant impacts on POI. Several studies indicated that low-carbohydrate and high-protein diets can lead to improvements in metabolism in humans (Foster *et al*, 2003; Huhmann *et al*, 2018; de Castro *et al*, 2019). In the context of the reproduction study, the positive correlation between dietary protein intake and outcomes of infertility treatment in women was also identified (Nassan *et al*, 2018). In contrast, the negative correlation between dietary protein intake and antral follicle counts was also observed in women receiving infertility treatment (Souter *et al*, 2017). In addition, a recent study on sexually immature female mice (4-week-old) revealed that the restricted protein intake resulted in an augmentation of oocyte number and fertility (Zhuo *et al*, 2019). As the experiment started with 4-week-old mice which are sexually immature, the dietary restriction may affect the development of the ovary. Notably, most of these studies measured or manipulated the proportion of crude proteins instead of the amount of specific amino acid in the diet.

In the current study, we identified that dietary restriction of BCAA specifically induced POI in mice. In advance, the dietary supplement of BCAA protected the mice from ROS-related POI. Based on the published data (Mardinoglu *et al*, 2018) from a human study, we also found that 14-day low-carbohydrate and high-protein diets increased the serum concentration of BCAA and decreased ceramide (Appendix Fig S4A–E). We hypothesized that restoring the proper BCAA level with a dietary supplement of BCAA can be developed as a therapy to prevent ovarian dysfunction in early-stage POI patients. We are going to test the hypothesis in the future with a clinical trial.

 © 2023 The Authors

In contrast to preventing further loss of ovarian function in the early stages, identifying therapies for restoring ovarian function in the late stages of POI is significantly more difficult. We found that the ceramide-treated KGN cell showed impaired function. This result can be used to build cellular models for phenotypic screens or mechanism studies to identify targeted therapies for BCAA deficiency-induced POI.

Mechanistically, many studies have described the connections among amino acid metabolism, FGF21, adiponectin, and ceramide (Markova *et al*, 2017; Andrade *et al*, 2021; Jachthuber Trub *et al*, 2021). The FGF21-adiponectin axis has been discovered as important factor for female fertility (Zhuo *et al*, 2019). Especially, adiponectin has also been identified as an important regulator of ceramide metabolism in several mouse models (Holland *et al*, 2011, 2013, 2017; Field *et al*, 2020). With great interest, we explored the role of adiponectin in POI. Unfortunately, we did not discover dramatic changes in serum concentration for adiponectin in either POI patients or mice fed a low BCAA diet (Appendix Fig S5A and B). Therefore, adiponectin may not be directly related to the development of BCAA insufficiencies-induced POI.

The use of a BCAA restriction-based diet as a therapy to lose weight is a topic of scientific research and is an attractive idea to the general public. A large-scale, long-term clinical trial of dietary BCAA reduction, with the change of body weight as one of the primary outcomes, has been designed and aims to recruit 132 obese patients as participants (NCT04424537). The results of our study do not hurt the potential for the low BCAA diet's clinical application, since the experiments were performed on lean mice. However, clinicians need to educate their patients properly and design the therapies carefully to avoid side effects.

Materials and Methods

Human subjects

The experiments were performed according to the principles of the WMA Declaration of Helsinki and Department of Health and Human Services Belmont Report. Whole blood was obtained from patients undergoing premature ovarian insufficiency in outpatient at Fudan University OB&GYN Hospital or Shandong University under protocol 2019-106. Written informed consent was obtained from each individual donating tissue and samples were anonymized and handled according to the ethical guidelines set forth by the Fudan University OB&GYN Hospital ethical committee. POI was defined as oligo/amenorrhea for at least 4 months with an elevated FSH level > 25 IU/l on two occasions > 4 weeks apart. All the enrolled patients were carefully consulted. Patients with autoimmune diseases or a family history of reproductive diseases were excluded from the study. No abnormality was revealed in the genetic and immunological tests. None of the POI patients were exposed to any kinds of endocrine therapies for POI when they were recruited, except the patients for ROS detection. The blood of the control group was collected during menstruation, while the blood of POI patients was collected randomly.

Reagents

Reagents involved in this study were listed in Dataset EV12.

Mouse experiments

All animal experiments were performed according to procedures approved by the Fudan University ethical committee (IDM2021006b). Mice were maintained under a 12 h light/12 h dark cycle at constant temperature (23°C) with free access to food and water. Wild-type C57B6/J mice were obtained from Jiesijie lab. The 8-week-old male mice were used. The mice were divided into different groups randomly. The investigators were blind to the labels of the groups. No mice were excluded from the experiments. The mice were on a special diet were fed with a low BCAA diet (Trophic Animal Feed High-tech Co., Ltd) for 4 weeks before mating or sacrifice. Toxin-induced POI mouse model was established by injection of Tripterygium Glycosides (TG) at 50 mg/kg from YUAN DA FEI YUN PHARMACY once per day for 5 weeks. The serum was collected via the tail vein. The liver and ovary were collected after the mice were sacrificed. BCAA supplementation was performed by dissolving BCAA in water with 10 mM Tween 80 at the dose of 25 g/l with a ratio of 2:1:1 (Leu:Ile:Val) (Hong *et al*, 2017). Concentration of three amino acids were 1.25% (Leu), 0.625% (Ile), and 0.625% (Val). Ceramide treatment was performed by dissolving the ceramide mixture in water at a dose of 30 mg/kg. To inhibit the effects of ceramide, the mice were injected with 0.5 mg/kg of myriocin intraperitoneally every day. All the mice were free to drink water and drug beverages were renewed each day.

ELISA

Blood samples were centrifuged at 3,000 rpm for 15 min to collect serum. Samples were kept at −80°C. The cell culture medium was collected at 1,500 rpm for 10 min to remove the cell residuals. FGF21, GDF15, FSH, E2, AMH, SOD, GSH-Px, and MDA levels were measured by ELISA kit according to the manufacturer's instructions.

Lipid and metabolite extraction

The extraction method was modified from a published article (Huang *et al*, 2020), and briefly described here. Fifty microliters of serum were collected from either POI patients or healthy ones. The serum was first centrifuged at 14,000 *g* for 10 min at 4°C, and the supernatant was decanted into a new glass centrifuge tube. Two hundred microliters of water, 1 ml of methanol, and 5 ml of MTBE were added to the glass centrifuge tube following a 1 min vortexing. Once incubating the mixture on a rotator for 1 h at room temperature, 1.5 ml more water were added to the mixture and vortexed for another 1 min. The mixture was then centrifuged at 1,000 *g* for 10 min at 4°C to separate the two phases; the upper phase containing nonpolar lipids and the lower phase containing aqueous metabolites. Two phases were separated, collected, and dried using a SpeedVac at room temperature. The dried lipids or metabolites were stored at −80°C until analyzed by LC–MS.

Untargeted lipidomics

The nonpolar lipids were reconstituted using 200 µl of 2-propanol:acetonitrile:water (*v:v:v* 30:65:5). Five microliters of reconstituted sample were injected into the LC–MS. The untargeted lipidomics method was modified from a published method (Breitkopf *et al*,

© 2023 The Authors

2017) that used C30 column (Acclaim C30, 3 µm, 2.1 × 150 mm). The LC method used two elution solutions; buffer A (60% acetonitrile and 40% water with 0.1% formic acid and 10 mM ammonium formate) and buffer B (90% 2-propanol and 10% acetonitrile with 0.1% formic acid and 10 mM ammonium formate). The 0.2 ml/min LC gradient was started from 0 to 1.5 min, 32% B, 4 min, 45% B, 5 min, 52% B, 8 min, 58% B, 11 min, 66% B, 14 min, 70% B, 18 min, 75% B, 21 to 25 min, 97% B, 25 to 32 min 32% B. The samples were acquired by an Orbitrap Exploris 480 (Thermo Fisher Scientific) using a polarity switching approach with DDA mode. All the lipidomics. RAW files were processed on *LipidSearch* 4.0 (Thermo Fisher Scientific) for lipid identification.

We profiled our lipidomic results in a highly confident manner which is following a strict feature selection and identification filtering rule.

The identification of untargeted lipidomic results is following certain criteria. Once the RAW files were acquired from LC–MS/MS, we searched the lipid feature using LipidSearch with such parameters:

1 Precursor tolerance 5 ppm and product tolerance 5 ppm.
2 The relative intensity threshold for the parent ion is 0.01 and production is 1.0%.
3 The peak extraction for m/z is ±5 ppm and ±0.5 min for the RT.
4 The S/N threshold is 3 for the MS^1 and 1 for the MS^2 peaks.

To ensure positive lipid identification, we filtered the lipids based on top-ranked features whose m-score is higher than 5.0 and whose C-score is higher than 2.0. The lipid features were also selected under FA priority and ID quality including A/B/C scores.

Targeted metabolomics

The aqueous metabolites were reconstituted using 100 µl of acetonitrile:water (v:v 50:50). Five microliters of reconstituted sample were injected into the LC–MS. The targeted metabolomics method was modified from a published protocol (Yuan *et al*, 2012) that used an amide HILIC column (XBridge Amide 3.5 µm, 4.6 × 100 mm). The LC method used two elution solutions; buffer A (95% water and 5% acetonitrile with 20 mM of ammonium hydroxide and 20 mM of ammonium acetate, pH 9.0) and buffer B (acetonitrile). The 0.25 ml/min LC gradient was started from 0 to 0.1 min, 85% B, 3.5 min, 32% B, 12 min, 2% B, 16.5 min, 2% B, 17 to 16 min, 85% B. The samples were acquired by a QTRAP 5500+ (AB Sciex) using a polarity switching approach which referred from a published MRM list containing 297 transitions. The LC–MS/MS peak integration was performed on MultiQuant (AB Sciex) to obtain the metabolomics spreadsheet.

Metabolomic and lipidomic data analyses

The MetaboAnalyst 5.0 and LINT-web website was utilized to analyze metabolomic and lipidomics results. In brief, the data were normalized to the median value, log-transformed, and auto-scaled. Then, the relative abundance of individual metabolites/lipids and the mean abundance of ceramides was calculated based on the processed data. The details of how these tools work were described in the literature (Pang *et al*, 2020) and (Li *et al*, 2021a). The raw data were deposited at Metabolights as MTBLS6249 (lipid) and MTBLS6250 (metabolites).

Proteomics analysis

For high-abundance protein depletion of plasma, samples were first incubated in the High-Select Top14 Abundant Protein Depletion Mini Spin Columns (Thermo Fisher Scientific) at 25°C for 2 h according to the manufacturer's instructions. The filtrates were vacuum-dried and redissolved in 8 M urea and 100 mM pH 8.0 Tris–HCl. Samples were reduced with 5 mM DTT at 37°C for 30 min. Then samples were alkylated with 15 mM IAM at 25°C for 45 min in dark. The excess IAM was quenched with DTT. The samples were digested with 1/50 trypsin at 37°C overnight. The resulting peptides were acidified, desalted using homemade R3 micro-columns, and vacuum-dried completely.

To generate data-dependent acquisition (DDA) library, peptides were prefractionated on a Dionex UltiMate 3000 HPLC system (Thermo Fisher Scientific) using a C18 column (3 µm, 2 × 150 mm, Phenomenex, USA). HPLC solvent A was 10 mM NH_4HCO_3, solvent B was 10 mM NH_4HCO_3 in 80% ACN. Peptides from each sample were mixed (a total of 220 µg), dried, and then dissolved with 10 mM NH_4HCO_3. The mixture was separated by a linear gradient (5–40% in 25 min and 40–100% in 5 min) with a flow rate of 200 nl/min. Thirty fractions were mixed into 15 samples and vacuum-dried completely.

LC–MS/MS analysis was performed using an EASY-nLC 1200 system (Thermo Fisher Scientific) coupled to an Orbitrap Fusion Lumos mass spectrometer (Thermo Fisher Scientific). Samples were resuspended with 1% FA and iRT peptides (Biognosys) were added prior to MS analysis. Peptides were analyzed using a homemade C18 analytical column (75 µm i.d. × 25 cm, ReproSil-Pur 120 C18-AQ, 1.9 µm) (Dr. Maisch GmbH). The mobile phases consisted of Solvent A (0.1% formic acid) and Solvent B (0.1% formic acid in 80% ACN). The peptides were eluted using the following gradient: 2–5% B in 2 min, 5–35% B in 100 min, 35–44% B in 6 min, 44–100% B in 3 min, 100% B for 10 min, at a flow rate of 200 nl/min.

For DDA experiments, the resolution of full MS scans was set as 60,000 at m/z 200. AGC target was set as 4e5 with a maximum injection time of 50 ms. The scan range was set as 350–1,600 m/z.

For MS2, the AGC target was set as 200% with a resolution of 15,000 and a maximum injection time of 22 ms. The width of the isolation window was set as 1.3 m/z. The NCE was set as 30%. The cycle time was set as 3 s.

The data-independent acquisition (DIA) analysis was set as three full MS scans. Each full MS scan was followed by 20 MS2 windows. The first 20 windows were set from 350 to 550 m/z. The second 20 windows were set from 550 to 750 m/z. The third was set as 10 windows from 750 to 850 m/z, 5 windows from 850 to 950 m/z, and 5 windows from 950 to 1,200 m/z. The resolution of full MS scans was set as 120,000 at m/z 200, full MS AGC target was 100% with a maximum injection time of 50 ms and the scan range was set as 350–1,200. The resolution of MS2 was set as 30,000 with a maximum injection time of 54 ms and the NCE was set as 32%.

DDA data were processed using Protein Discoverer (version 1.4, Thermo Fisher Scientific) with Mascot (version 2.7, Matrix Science). The database was UniProt human protein database (75,004 entries) combined with the sequences of Biognosys iRT peptides. The mass tolerances were 10 ppm for precursor and 0.05 Da for fragment ions. Up to two missed cleavages were

 © 2023 The Authors

allowed. Carbamidomethylation (CAM) on cysteine was chosen as a fixed modification. Acetylation on protein N-terminal and oxidation on methionine were chosen as variable modifications.

DIA data analysis was performed using Spectronaut (version 14.3, Biognosys). High-precision iRT calibration was used. The library was generated by importing the search results from Proteome Discoverer using the default settings. DIA data were analyzed using default settings disabling the PTM localization filter. Mass tolerance/accuracy for precursor and fragment identification was set to default settings. Up to six fragments were employed for library generation. FDR at peptide and protein level was set to 1% using a mutated decoy model. To match DIA data to the spectral library, the applied mass and retention time tolerances were dynamic based on the m/z of the targeted ion and the retention time of the scan. The calibration was done for each run individually. Default settings for quantification at the MS1 level were employed for quantification. The raw data were deposited at iProX (https://www.iprox.cn/, accession ID: IPX0005189000).

RNA-seq assay

Total RNA was extracted from normal and pathological tissues with the TRIzol reagent (Invitrogen). RNAs were then reversely transcribed with oligo(dT) primers. RNA-seq libraries for expression analysis were constructed using KAPA RNA HyperPrep Kit KR1350 v1.16 according to the vendor's protocol and paired-end 2 × 150 bp reads were sequenced using the Illumina HiSeq platform. The data were aligned and quantified by HISAT2 (Kim *et al*, 2019). The raw data were deposited to GEO (GSE215358).

Single nuclei RNA-seq

The ovaries were collected from the mice and immediately froze in liquid nitrogen. Before the Single nuclei RNA-seq (sNuc-seq) started, the ovaries were minced and homogenized in a nuclei extraction buffer (Tris 10 mM, Tween-20 0.03%, NaCl 146 mM, $CaCl_2$ 1 mM, $MgCl_2$ 21 mM, and BSA 0.01%). The nuclei were immediately loaded on the 10× Chromium controller (10× Genomics) with Single Cell 3′ v3.1 chemistry according to the manufacturer's protocol after FACS purification. For each sample, ~ 10,000 nuclei were loaded in one channel of a Chromium Chip (10× Genomics). The cDNA generation and library preparation were performed according to the manufacturer's protocol and sequenced using the Illumina HiSeq platform. The data was initially processed by Cell Ranger and analyzed by Seurat v4.0.

Quantitative RT-PCR

TRIzol (Thermo Fisher) was used for total tissue and RNA isolation. Extracted RNA (500 ng) was converted into cDNA using the PrimeScript™ RT reagent Kit (Takara). Quantitative RT-PCR (qRT-PCR) was performed using an Applied Biosystems QuantStudio 5 and SYBR Green PCR Master Mix (Applied Biosystems). Fold change was determined by comparing target gene expression with the reference gene *36b4* (Forward: GAGGAATCAGATGAGGATATGGGA; Reverse: AAGCAGGCTGACTTGGTTGC). The primer sequences of *Fgf21* were: Forward: ACTGAAGCCCACCTGGAGAT; Reverse: AGGCTTTGACACCCAGGATT.

Cell culture

The KGN cells (Procell, STR tested) were maintained in DMEM/F12 medium (HyClone) with 10% FBS (Gibco) and 1% penicillin/streptomycin mixture (Gibco). 10^5 cells were seeded in a 24-well plate for experiments. The KGN cells were treated with ceramides (MCE) or DMSO for 48 h and stimulated with FSH (MCE) for 24 h, before the medium was collected to measure the concentration of E2 by ELISA. The E2 concentration was normalized to the protein concentration of the cells, for which the cells were lysed by NP40 buffer and measured with a BCA kit (Thermo Fisher Scientific). Cell viability was measured with Cell Counting Kit-8 (CCK-8; Yeasen) according to the manufacturer's instructions. Briefly, cells were seeded into a 96-well plate at a density of 1,000 cells per well. Cells were examined at 48 h w/o ceramide treatment. In brief, CCK-8 (10%) was added to the wells. After an incubation of 1 h at 37°C, the absorbance was measured at 450 nm with Microplate Reader Infinite® F50 (Tecan). The Annexin V assay was performed with an apoptosis kit (Servicebio, G1510-50T) and Agilent NovoCyte 3130 following the manufacturer's guideline.

Histology

The right ovaries were collected and fixed in 4% paraformaldehyde (DINGGUO) after all the mice were sacrificed. Each ovary was embedded in paraffin and sectioned into three slides within 3 mm from the maximum cross section and mounted on glass slides. Samples were dewaxed using Xylene (Sinopharm Chemical Reagent Co., Ltd) for 20 min twice, 100% ethanol (Sinopharm Chemical Reagent Co., Ltd) for 5 min twice, 75% ethanol for 5 min, and rinsing with tap water. Sections were stained with Hematoxylin solution (ServiceBio) for 3–5 min, and rinsed with tap water. Then sections were treated with Hematoxylin Differentiation solution (ServiceBio) and Hematoxylin Scott Tap Bluing (ServiceBio), rinse with tap water, respectively. Sections were dehydrated using 85% ethanol for 5 min and 95% ethanol for 5 min, stained with Eosin dye (ServiceBio) for 5 min. Then sections were dehydrated using 100% ethanol for 5 min three times and xylene for 5 min twice. Finally, sections were sealed with neutral gum (Sinopharm Chemical Reagent Co., Ltd).

All the ovarian follicles' images were taken by OLYMPUS BX53F Optical Microscope. Follicles with oocyte nucleus were recognized to avoid repeated counts of the same follicle. The follicles were classified as primordial, primary, secondary, antral follicle, and atresia follicle with the following protocol. Primordial follicle: an oocyte surrounded by one layer of flattened granulosa cells; primary follicle: one to two complete layers of cuboidal granulosa cells surrounding the oocyte; secondary follicle: more than two layers of cuboidal granulosa cells surrounding the oocyte; antral follicle: an oocyte surrounded by multiple layers of cuboidal granulosa cells with a cumulus oophorus and antral spaces; and atretic follicle: a follicle in a degenerative process with a formless nucleus.

Metabolic profile

The energy expenditure was measured by Promethion Comprehensive Lab Animal Monitoring System (CLAMS, Sable Systems International, NV, USA) housed within a temperature-controlled

The paper explained

Problems
Premature ovarian insufficiency (POI) is a disease featured by early menopause before 40 years of age with follicle-stimulating hormone > 25 U/l. Consequently, women with POI suffer from subfertility and are susceptible to estrogen deficiency-related aging symptoms in the bone, cardiovascular system, and central nervous system. Hormone replacement therapy (HRT) can certainly alleviate these symptoms, though the therapies to prevent or cure POI itself are still absent. A better understanding of its pathogenesis is important to develop specific therapies, other than HRT, to prevent or cure POI.

Results
In this study, we investigated the metabolic changes of POI patients who had never been exposed to HRT by liquid chromatography–mass spectrometry-based metabolomics. We found low serum branch chain amino acid (BCAA) levels in these patients, which was validated in an independent cohort collected in a different center. With multiple models, we validated that BCAA abundance regulates ovarian function and fertility via the effects of the ceramide-reactive oxygen species (ROS) axis on ovarian granulosa cells. Additionally, dietary supplementation with BCAA protects ovaries from ROS-induced POI in mice.

Impacts
Our study reveals that restoring the proper BCAA level with a dietary supplement of BCAA can be developed as a therapy to prevent ovarian dysfunction in early-stage POI patients. This hypothesis should be tested with clinical trials in the future. In addition, clinicians need to educate their patients on low BCAA diet properly and design the therapies carefully to avoid side effects.

environmental chamber at Fudan University. Female mice on control or low BCAA diet from 6 weeks were housed in the room for CLAMS 24 h before the experiment started. The CLAMS experiment was performed for 48 h according to the manufacturer's guidelines. The data analysis was performed in a similar procedure as described in the literature (Li *et al*, 2021b). The energy content of the diet was measured by a bomb calorimeter (IKA, Guangdong, China) according to the manufacturer's guidelines.

Statistics

GSEA was performed according to its guideline using the default setting (Mootha *et al*, 2003; Subramanian *et al*, 2005). The significant changes from GSEA were defined as the absolute value of normalized enrichment score (NES) > 1, $P < 0.05$, and $q < 0.25$. The P- and r-value for the correlation analysis were calculated by the nonparametric Spearman test. The contingency test was used for the analysis of the pregnancy rate. Paired student t-test was used for the analysis of the data from patients on a high protein low carbohydrate diet. Student t-test with 2-way ANOVA correction was used for the rest of the data in this study.

Data availability

The raw metabolomics and lipidomics data were deposited at Metabolights as MTBLS6249 (lipid) and MTBLS6267 (metabolites). The raw proteomics data were deposited at iProX (https://www.iprox.cn/, accession ID: IPX0005189000). The raw RNA-seq data were deposited at GEO (GSE215358).

Expanded View for this article is available online.

Acknowledgments
This work was supported by MOST 2020YFA0803601, 2018YFA0801300, NSFC 32071138, and SKLGE-2118 to JL, NSFC 92057115, Shanghai Sailing Program 20YF1402600, MOST 2020YFA0803800, and 2019YFA0801900 to HH, NSFC 82171633 and Natural Science Foundation of Shanghai 20ZR1408800 to CG, NSFC 82271670, Natural Science Foundation of Shanghai 19ZR1406800 and 22ZR1408700 to BL, IDH1322092/010 to SH, and NSFC 8202201 and 81970740 to XX. We thank Dr. Xiaofei Yu, Dr. Wei Yu, and Dr. Xiaohui Wu (Fudan University) for their insightful discussion. The data for the human study of the low carbohydrate and high protein diet was kindly provided by Dr. Fredrik Bäckhed (University of Gothenburg). We thank Single Cell Quantitative Metabolomics and Lipidomics Core Facility of IMIB at Fudan University for LC–MS/MS analysis.

Author contributions
Xiao Guo: Conceptualization; data curation; formal analysis; validation; investigation; visualization; methodology; writing—original draft; project administration; writing—review and editing. **Yuemeng Zhu:** Conceptualization; data curation; formal analysis; validation; investigation; visualization; methodology; writing—original draft; project administration; writing—review and editing. **Lu Guo:** Conceptualization; data curation; formal analysis; validation; investigation; visualization; methodology; writing—original draft; writing—review and editing. **Yiwen Qi:** Conceptualization; data curation; formal analysis; validation; investigation; visualization; methodology; writing—original draft; writing—review and editing. **Xiaocheng Liu:** Data curation; formal analysis; validation; investigation; visualization; methodology; writing—original draft; writing—review and editing. **Jinhui Wang:** Data curation; formal analysis; validation; investigation; visualization; methodology. **Jiangtao Zhang:** Data curation; formal analysis; investigation; methodology; writing—original draft; writing—review and editing. **Linlin Cui:** Formal analysis; investigation; methodology; writing—original draft; writing—review and editing. **Yueyang Shi:** Formal analysis; investigation; methodology; writing—original draft; writing—review and editing. **Qichu Wang:** Formal analysis; investigation; methodology; writing—original draft; writing—review and editing. **Guangxing Lu:** Data curation; formal analysis; investigation; methodology; writing—original draft; writing—review and editing. **Cenxi Liu:** Data curation; formal analysis; investigation; methodology; writing—original draft; writing—review and editing. **Yilian Liu:** Data curation; formal analysis; investigation; methodology; writing—original draft; writing—review and editing. **Tao Li:** Formal analysis; investigation; methodology; writing—original draft; writing—review and editing. **Shangyu Hong:** Conceptualization; formal analysis; investigation; writing—original draft; writing—review and editing. **Yingying Qin:** Data curation; formal analysis; investigation; methodology; writing—original draft; writing—review and editing. **Xuelian Xiong:** Data curation; formal analysis; funding acquisition; investigation; methodology; writing—original draft; writing—review and editing. **Hao Wu:** Data curation; formal analysis; investigation; methodology; writing—original draft; writing—review and editing. **Huang Lin:** Data curation; formal analysis; investigation; methodology; writing—original draft; writing—review and editing. **He Huang:** Conceptualization; data curation; formal analysis; supervision; funding acquisition; validation; investigation; visualization; methodology; writing—original draft; writing—review and editing. **Chao Gu:** Conceptualization; data curation; formal analysis; supervision; funding acquisition; validation;

© 2023 The Authors

investigation; visualization; methodology; writing—original draft; writing—review and editing. **Bin Li:** Conceptualization; data curation; formal analysis; supervision; funding acquisition; validation; investigation; visualization; methodology; writing—original draft; project administration; writing—review and editing. **Jin Li:** Conceptualization; data curation; formal analysis; supervision; funding acquisition; investigation; methodology; writing—original draft; project administration; writing—review and editing.

Disclosure and competing interests statement

The authors declare that they have no conflict of interest.

References

Andrade ML, Gilio GR, Perandini LA, Peixoto AS, Moreno MF, Castro E, Oliveira TE, Vieira TS, Ortiz-Silva M, Thomazelli CA *et al* (2021) PPARgamma-induced upregulation of subcutaneous fat adiponectin secretion, glyceroneogenesis and BCAA oxidation requires mTORC1 activity. *Biochim Biophys Acta Mol Cell Biol Lipids* 1866: 158967

Armeni E, Paschou SA, Goulis DG, Lambrinoudaki I (2021) Hormone therapy regimens for managing the menopause and premature ovarian insufficiency. *Best Pract Res Clin Endocrinol Metab* 35: 101561

Bachelot A, Rouxel A, Massin N, Dulon J, Courtillot C, Matuchansky C, Badachi Y, Fortin A, Paniel B, Lecuru F *et al* (2009) Phenotyping and genetic studies of 357 consecutive patients presenting with premature ovarian failure. *Eur J Endocrinol* 161: 179 – 187

Boulet MM, Chevrier G, Grenier-Larouche T, Pelletier M, Nadeau M, Scarpa J, Prehn C, Marette A, Adamski J, Tchernof A (2015) Alterations of plasma metabolite profiles related to adipose tissue distribution and cardiometabolic risk. *Am J Physiol Endocrinol Metab* 309: E736 – E746

Breitkopf SB, Ricoult SJH, Yuan M, Xu Y, Peake DA, Manning BD, Asara JM (2017) A relative quantitative positive/negative ion switching method for untargeted lipidomics via high resolution LC-MS/MS from any biological source. *Metabolomics* 13: 30

de Castro MBT, Cunha DB, Araujo MC, Bezerra IN, Adegboye ARA, Kac G, Sichieri R (2019) High protein diet promotes body weight loss among Brazilian postpartum women. *Matern Child Nutr* 15: e12746

Chapman C, Cree L, Shelling AN (2015) The genetics of premature ovarian failure: current perspectives. *Int J Womens Health* 7: 799 – 810

Cosentino RG, Churilla JR, Josephson S, Molle-Rios Z, Hossain MJ, Prado WL, Balagopal PB (2021) Branched-chain amino acids and relationship with inflammation in youth with obesity: a randomized controlled intervention study. *J Clin Endocrinol Metab* 106: 3129 – 3139

Cummings NE, Williams EM, Kasza I, Konon EN, Schaid MD, Schmidt BA, Poudel C, Sherman DS, Yu D, Arriola Apelo SI *et al* (2018) Restoration of metabolic health by decreased consumption of branched-chain amino acids. *J Physiol* 596: 623 – 645

Field BC, Gordillo R, Scherer PE (2020) The role of ceramides in diabetes and cardiovascular disease regulation of ceramides by adipokines. *Front Endocrinol (Lausanne)* 11: 569250

Foster GD, Wyatt HR, Hill JO, McGuckin BG, Brill C, Mohammed BS, Szapary PO, Rader DJ, Edman JS, Klein S (2003) A randomized trial of a low-carbohydrate diet for obesity. *N Engl J Med* 348: 2082 – 2090

He X, Zhang Y (2022) Protective effect of amino acids on the muscle injury of aerobics athletes after endurance exercise based on CT images. *J Healthc Eng* 2022: 5961267

Hernandez-Corbacho MJ, Canals D, Adada MM, Liu M, Senkal CE, Yi JK, Mao C, Luberto C, Hannun YA, Obeid LM (2015) Tumor necrosis factor-alpha (TNFalpha)-induced ceramide generation via ceramide synthases regulates loss of focal adhesion kinase (FAK) and programmed cell death. *J Biol Chem* 290: 25356 – 25373

Holland WL, Miller RA, Wang ZV, Sun K, Barth BM, Bui HH, Davis KE, Bikman BT, Halberg N, Rutkowski JM *et al* (2011) Receptor-mediated activation of ceramidase activity initiates the pleiotropic actions of adiponectin. *Nat Med* 17: 55 – 63

Holland WL, Adams AC, Brozinick JT, Bui HH, Miyauchi Y, Kusminski CM, Bauer SM, Wade M, Singhal E, Cheng CC *et al* (2013) An FGF21-adiponectin-ceramide axis controls energy expenditure and insulin action in mice. *Cell Metab* 17: 790 – 797

Holland WL, Xia JY, Johnson JA, Sun K, Pearson MJ, Sharma AX, Quittner-Strom E, Tippetts TS, Gordillo R, Scherer PE (2017) Inducible overexpression of adiponectin receptors highlight the roles of adiponectin-induced ceramidase signaling in lipid and glucose homeostasis. *Mol Metab* 6: 267 – 275

Hong CR, Lee GW, Paik HD, Chang PS, Choi SJ (2017) Nanosuspended branched chain amino acids: the influence of stabilizers on their solubility and colloidal stability. *Food Sci Biotechnol* 26: 573 – 579

Huang TT, Matsuyama HJ, Tsukada Y, Singhvi A, Syu RT, Lu Y, Shaham S, Mori I, Pan CL (2020) Age-dependent changes in response property and morphology of a thermosensory neuron and thermotaxis behavior in Caenorhabditis elegans. *Aging Cell* 19: e13146

Huhmann K (2020) Menses requires energy: a review of how disordered eating, excessive exercise, and high stress Lead to menstrual irregularities. *Clin Ther* 42: 401 – 407

Huhmann MB, Yamamoto S, Neutel JM, Cohen SS, Ochoa Gautier JB (2018) Very high-protein and low-carbohydrate enteral nutrition formula and plasma glucose control in adults with type 2 diabetes mellitus: a randomized crossover trial. *Nutr Diabetes* 8: 45

Ishizuka B (2021) Current understanding of the etiology, symptomatology, and treatment options in premature ovarian insufficiency (POI). *Front Endocrinol (Lausanne)* 12: 626924

Jachthuber Trub C, Balikcioglu M, Freemark M, Bain J, Muehlbauer M, Ilkayeva O, White PJ, Armstrong S, Ostbye T, Grambow S *et al* (2021) Impact of lifestyle intervention on branched-chain amino acid catabolism and insulin sensitivity in adolescents with obesity. *Endocrinol Diabetes Metab* 4: e00250

Karusheva Y, Koessler T, Strassburger K, Markgraf D, Mastrototaro L, Jelenik T, Simon MC, Pesta D, Zaharia OP, Bodis K *et al* (2019) Short-term dietary reduction of branched-chain amino acids reduces meal-induced insulin secretion and modifies microbiome composition in type 2 diabetes: a randomized controlled crossover trial. *Am J Clin Nutr* 110: 1098 – 1107

Kim D, Paggi JM, Park C, Bennett C, Salzberg SL (2019) Graph-based genome alignment and genotyping with HISAT2 and HISAT-genotype. *Nat Biotechnol* 37: 907 – 915

Lambrinoudaki I, Paschou SA, Lumsden MA, Faubion S, Makrakis E, Kalantaridou S, Panay N (2021) Premature ovarian insufficiency: a toolkit for the primary care physician. *Maturitas* 147: 53 – 63

Li FS, Song J, Zhang YK, Wang SK, Wang JH, Lin L, Yang CY, Li P, Huang H (2021a) LINT-web: a web-based lipidomic data mining tool using intra-Omic integrative correlation strategy. *Small Methods* 5: 2100206

Li J, Li E, Czepielewski RS, Chi J, Guo X, Han YH, Wang D, Wang L, Hu B, Dawes B *et al* (2021b) Neurotensin is an anti-thermogenic peptide produced by lymphatic endothelial cells. *Cell Metab* 33: 1449 – 1465.e6

Lin G, Lee PT, Chen K, Mao D, Tan KL, Zuo Z, Lin WW, Wang L, Bellen HJ (2018) Phospholipase PLA2G6, a parkinsonism-associated gene, affects Vps26 and Vps35, retromer function, and ceramide levels, similar to alpha-synuclein gain. *Cell Metab* 28: 605 – 618.e6

© 2023 The Authors

Lotta LA, Scott RA, Sharp SJ, Burgess S, Luan J, Tillin T, Schmidt AF, Imamura F, Stewart ID, Perry JR *et al* (2016) Genetic predisposition to an impaired metabolism of the branched-chain amino acids and risk of type 2 diabetes: a mendelian randomisation analysis. *PLoS Med* 13: e1002179

Luisi S, Orlandini C, Regini C, Pizzo A, Vellucci F, Petraglia F (2015) Premature ovarian insufficiency: from pathogenesis to clinical management. *J Endocrinol Invest* 38: 597 – 603

Mardinoglu A, Wu H, Bjornson E, Zhang C, Hakkarainen A, Rasanen SM, Lee S, Mancina RM, Bergentall M, Pietilainen KH *et al* (2018) An integrated understanding of the rapid metabolic benefits of a carbohydrate-restricted diet on hepatic steatosis in humans. *Cell Metab* 27: 559 – 571.e5

Markova M, Pivovarova O, Hornemann S, Sucher S, Frahnow T, Wegner K, Machann J, Petzke KJ, Hierholzer J, Lichtinghagen R *et al* (2017) Isocaloric diets high in animal or plant protein reduce liver fat and inflammation in individuals with type 2 diabetes. *Gastroenterology* 152: 571 – 585.e8

McGlacken-Byrne SM, Conway GS (2022) Premature ovarian insufficiency. *Best Pract Res Clin Obstet Gynaecol* 81: 98 – 110

Michalakis K, Coppack SW (2012) Primary ovarian insufficiency: relation to changes in body composition and adiposity. *Maturitas* 71: 320 – 325

Mootha VK, Lindgren CM, Eriksson KF, Subramanian A, Sihag S, Lehar J, Puigserver P, Carlsson E, Ridderstrale M, Laurila E *et al* (2003) PGC-1alpha-responsive genes involved in oxidative phosphorylation are coordinately downregulated in human diabetes. *Nat Genet* 34: 267 – 273

Nassan FL, Chiu YH, Vanegas JC, Gaskins AJ, Williams PL, Ford JB, Attaman J, Hauser R, Chavarro JE, Team ES (2018) Intake of protein-rich foods in relation to outcomes of infertility treatment with assisted reproductive technologies. *Am J Clin Nutr* 108: 1104 – 1112

Newgard CB, An J, Bain JR, Muehlbauer MJ, Stevens RD, Lien LF, Haqq AM, Shah SH, Arlotto M, Slentz CA *et al* (2009) A branched-chain amino acid-related metabolic signature that differentiates obese and lean humans and contributes to insulin resistance. *Cell Metab* 9: 311 – 326

Ottenlinger FM, Mayer CA, Ferreiros N, Schreiber Y, Schwiebs A, Schmidt KG, Ackermann H, Pfeilschifter JM, Radeke HH (2016) Interferon-Beta increases plasma ceramides of specific chain length in multiple sclerosis patients, unlike fingolimod or natalizumab. *Front Pharmacol* 7: 412

Owen BM, Bookout AL, Ding X, Lin VY, Atkin SD, Gautron L, Kliewer SA, Mangelsdorf DJ (2013) FGF21 contributes to neuroendocrine control of female reproduction. *Nat Med* 19: 1153 – 1156

Panay N, Anderson RA, Nappi RE, Vincent AJ, Vujovic S, Webber L, Wolfman W (2020) Premature ovarian insufficiency: an international menopause society White paper. *Climacteric* 23: 426 – 446

Pang Z, Chong J, Li S, Xia J (2020) MetaboAnalystR 3.0: toward an optimized workflow for global metabolomics. *Metabolites* 10: 186

Papathanassiu AE, Ko JH, Imprialou M, Bagnati M, Srivastava PK, Vu HA, Cucchi D, McAdoo SP, Ananieva EA, Mauro C *et al* (2017) BCAT1 controls metabolic reprogramming in activated human macrophages and is associated with inflammatory diseases. *Nat Commun* 8: 16040

Pedersen HK, Gudmundsdottir V, Nielsen HB, Hyotylainen T, Nielsen T, Jensen BA, Forslund K, Hildebrand F, Prifti E, Falony G *et al* (2016) Human gut microbes impact host serum metabolome and insulin sensitivity. *Nature* 535: 376 – 381

Purpera MN, Shen L, Taghavi M, Munzberg H, Martin RJ, Hutson SM, Morrison CD (2012) Impaired branched chain amino acid metabolism alters feeding behavior and increases orexigenic neuropeptide expression in the hypothalamus. *J Endocrinol* 212: 85 – 94

Rehnitz J, Messmer B, Bender U, Nguyen XP, Germeyer A, Hinderhofer K, Strowitzki T, Capp E (2022) Activation of AKT/mammalian target of rapamycin signaling in the peripheral blood of women with premature ovarian insufficiency and its correlation with FMR1 expression. *Reprod Biol Endocrinol* 20: 44

Richardson NE, Konon EN, Schuster HS, Mitchell AT, Boyle C, Rodgers AC, Finke M, Haider LR, Yu D, Flores V *et al* (2021) Lifelong restriction of dietary branched-chain amino acids has sex-specific benefits for frailty and life span in mice. *Nat Aging* 1: 73 – 86

Riva A, Falbo M, Passoni P, Polizzi S, Cattoni A, Nacinovich R (2021) High levels of physical activity in female adolescents with anorexia nervosa: medical and psychopathological correlates. *Eat Weight Disord* 27: 151 – 162

Samad N, Nguyen HH, Ebeling PR, Milat F (2020) Musculoskeletal health in premature ovarian insufficiency. Part two: bone. *Semin Reprod Med* 38: 289 – 301

Singhal G, Douris N, Fish AJ, Zhang X, Adams AC, Flier JS, Pissios P, Maratos-Flier E (2016) Fibroblast growth factor 21 has no direct role in regulating fertility in female mice. *Mol Metab* 5: 690 – 698

Souter I, Chiu YH, Batsis M, Afeiche MC, Williams PL, Hauser R, Chavarro JE, Team ES (2017) The association of protein intake (amount and type) with ovarian antral follicle counts among infertile women: results from the EARTH prospective study cohort. *BJOG* 124: 1547 – 1555

Stevenson JC, Collins P, Hamoda H, Lambrinoudaki I, Maas A, Maclaran K, Panay N (2021) Cardiometabolic health in premature ovarian insufficiency. *Climacteric* 24: 474 – 480

Strock NCA, De Souza MJ, Williams NI (2020) Eating behaviours related to psychological stress are associated with functional hypothalamic amenorrhoea in exercising women. *J Sports Sci* 38: 2396 – 2406

Subramanian A, Tamayo P, Mootha VK, Mukherjee S, Ebert BL, Gillette MA, Paulovich A, Pomeroy SL, Golub TR, Lander ES *et al* (2005) Gene set enrichment analysis: a knowledge-based approach for interpreting genome-wide expression profiles. *Proc Natl Acad Sci USA* 102: 15545 – 15550

Szegda KL, Whitcomb BW, Purdue-Smithe AC, Boutot ME, Manson JE, Hankinson SE, Rosner BA, Bertone-Johnson ER (2017) Adult adiposity and risk of early menopause. *Hum Reprod* 32: 2522 – 2531

Tournissac M, Vandal M, Tremblay C, Bourassa P, Vancassel S, Emond V, Gangloff A, Calon F (2018) Dietary intake of branched-chain amino acids in a mouse model of Alzheimer's disease: effects on survival, behavior, and neuropathology. *Alzheimers Dement (N Y)* 4: 677 – 687

Tsiligiannis S, Panay N, Stevenson JC (2019) Premature ovarian insufficiency and long-term health consequences. *Curr Vasc Pharmacol* 17: 604 – 609

Tucker EJ, Grover SR, Bachelot A, Touraine P, Sinclair AH (2016) Premature ovarian insufficiency: new perspectives on genetic cause and phenotypic Spectrum. *Endocr Rev* 37: 609 – 635

Vegetti W, Grazia Tibiletti M, Testa G, de Lauretis Y, Alagna F, Castoldi E, Taborelli M, Motta T, Bolis PF, Dalpra L *et al* (1998) Inheritance in idiopathic premature ovarian failure: analysis of 71 cases. *Hum Reprod* 13: 1796 – 1800

Webber L, Anderson RA, Davies M, Janse F, Vermeulen N (2017) HRT for women with premature ovarian insufficiency: a comprehensive review. *Hum Reprod Open* 2017: hox007

Woo CY, Baek JY, Kim AR, Hong CH, Yoon JE, Kim HS, Yoo HJ, Park TS, Kc R, Lee KU *et al* (2020) Inhibition of ceramide accumulation in podocytes by myriocin prevents diabetic nephropathy. *Diabetes Metab J* 44: 581 – 591

Xu M, Qi Q, Liang J, Bray GA, Hu FB, Sacks FM, Qi L (2013) Genetic determinant for amino acid metabolites and changes in body weight and insulin resistance in response to weight-loss diets: the preventing overweight using novel dietary strategies (POUNDS LOST) trial. *Circulation* 127: 1283 – 1289

© 2023 The Authors

Xu B, Li Z, Li S, Ke H, Zhang Q, Qin Y, Guo T (2022) Pathogenic variants in TSC2 might cause premature ovarian insufficiency through activated mTOR induced hyperactivation of primordial follicles. *Fertil Steril* 118: 1139 – 1149

Yang RX, Pan Q, Liu XL, Zhou D, Xin FZ, Zhao ZH, Zhang RN, Zeng J, Qiao L, Hu CX *et al* (2019) Therapeutic effect and autophagy regulation of myriocin in nonalcoholic steatohepatitis. *Lipids Health Dis* 18: 179

Yao T, Yan H, Zhu X, Zhang Q, Kong X, Guo S, Feng Y, Wang H, Hua Y, Zhang J *et al* (2022) Erratum. Obese Skeletal Muscle-Expressed Interferon Regulatory Factor 4 Transcriptionally Regulates Mitochondrial Branched-Chain Aminotransferase Reprogramming Metabolome. Diabetes 2022;71:2256–2271. *Diabetes* 72: 309

Yu D, Richardson NE, Green CL, Spicer AB, Murphy ME, Flores V, Jang C, Kasza I, Nikodemova M, Wakai MH *et al* (2021) The adverse metabolic effects of branched-chain amino acids are mediated by isoleucine and valine. *Cell Metab* 33: 905 – 922.e6

Yuan M, Breitkopf SB, Yang X, Asara JM (2012) A positive/negative ion-switching, targeted mass spectrometry-based metabolomics platform for bodily fluids, cells, and fresh and fixed tissue. *Nat Protoc* 7: 872 – 881

Zhenyukh O, Civantos E, Ruiz-Ortega M, Sanchez MS, Vazquez C, Peiro C, Egido J, Mas S (2017) High concentration of branched-chain amino acids promotes oxidative stress, inflammation and migration of human peripheral blood mononuclear cells via mTORC1 activation. *Free Radic Biol Med* 104: 165 – 177

Zhou M, Shao J, Wu CY, Shu L, Dong W, Liu Y, Chen M, Wynn RM, Wang J, Wang J *et al* (2019) Targeting BCAA catabolism to treat obesity-associated insulin resistance. *Diabetes* 68: 1730 – 1746

Zhuo Y, Hua L, Feng B, Jiang X, Li J, Jiang D, Huang X, Zhu Y, Li Z, Yan L *et al* (2019) Fibroblast growth factor 21 coordinates adiponectin to mediate the beneficial effects of low-protein diet on primordial follicle reserve. *EBioMedicine* 41: 623 – 635

License: This is an open access article under the terms of the Creative Commons Attribution License, which permits use, distribution and reproduction in any medium, provided the original work is properly cited.

Expanded View Figures

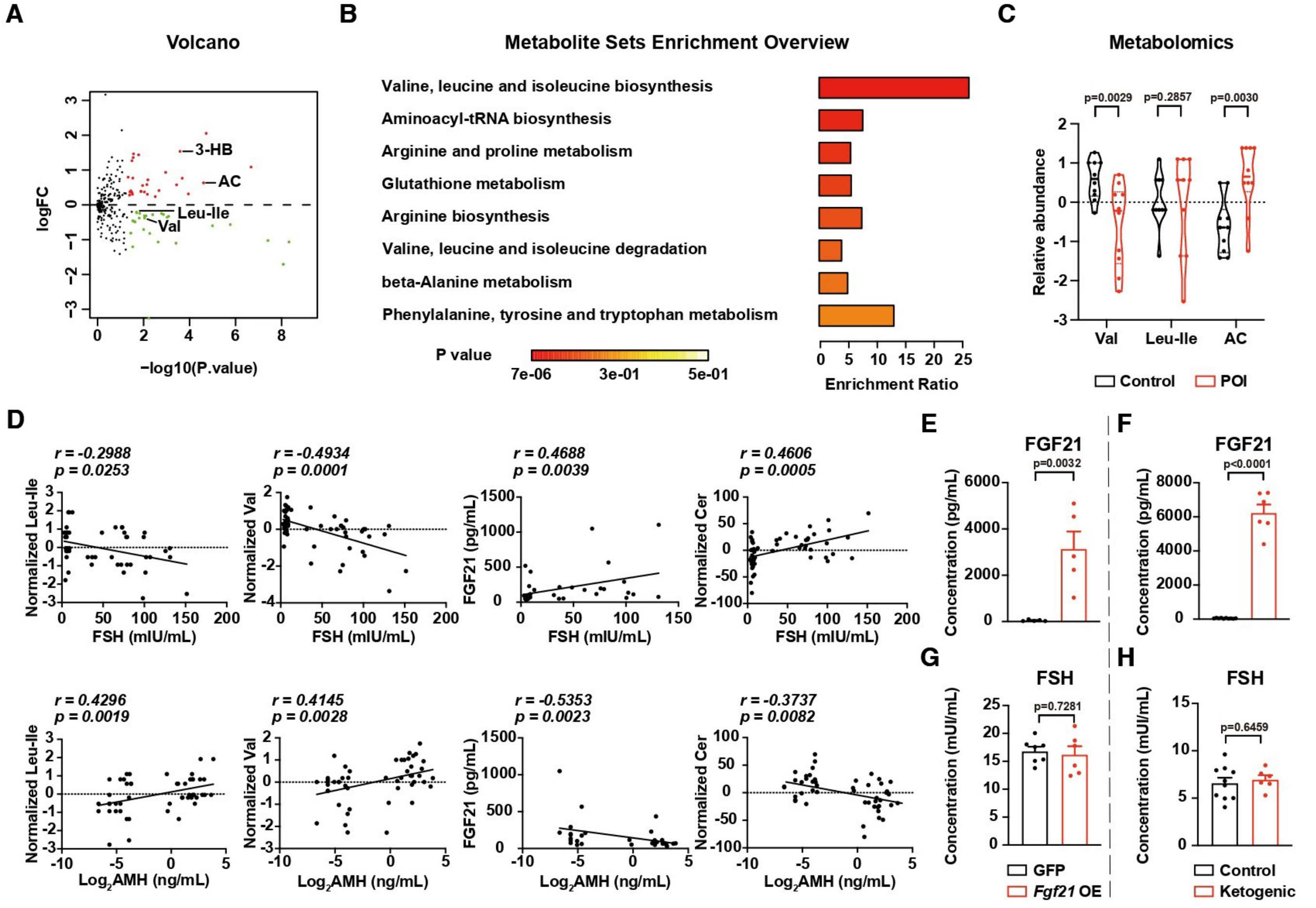

Figure EV1. Metabolic details of POI patients.

A Volcano plot showing the fold-change and *P*-value of metabolites. $N = 18$.

B Enrichment score and *P*-value of metabolites downregulated in POI patients. $N = 18$.

C Relative abundance of valine (Val), acetyl-carnitine (AC) but not leucine-isoleucine (Leu-Ile) in the serum of the Shandong Cohort. $N = 10$; Truncated violin plot, central band stands for median, and dotted lines stand for the upper quartile or the lower quartile of the data.

D The correlations between metabolites/FGF21 and clinical parameters. The *P* and *r* were calculated by the nonparametric Spearman test. $N = 56$.

E, F The concentration of FGF21 in mouse serum. (E) $N = 5$; (F) Control $N = 10$; Ketogenic diet, $N = 6$.

G, H The concentration of FSH in mouse serum. (G) Control, $N = 7$; *Fgf21* OE, $N = 6$; and (H) control, $N = 10$; Ketogenic diet, $N = 6$.

Data information: Error bars stand for SEM. The *P*-value was calculated by a two-tailed *t*-test with 2-way ANOVA correction.
Source data are available online for this figure.

 © 2023 The Authors

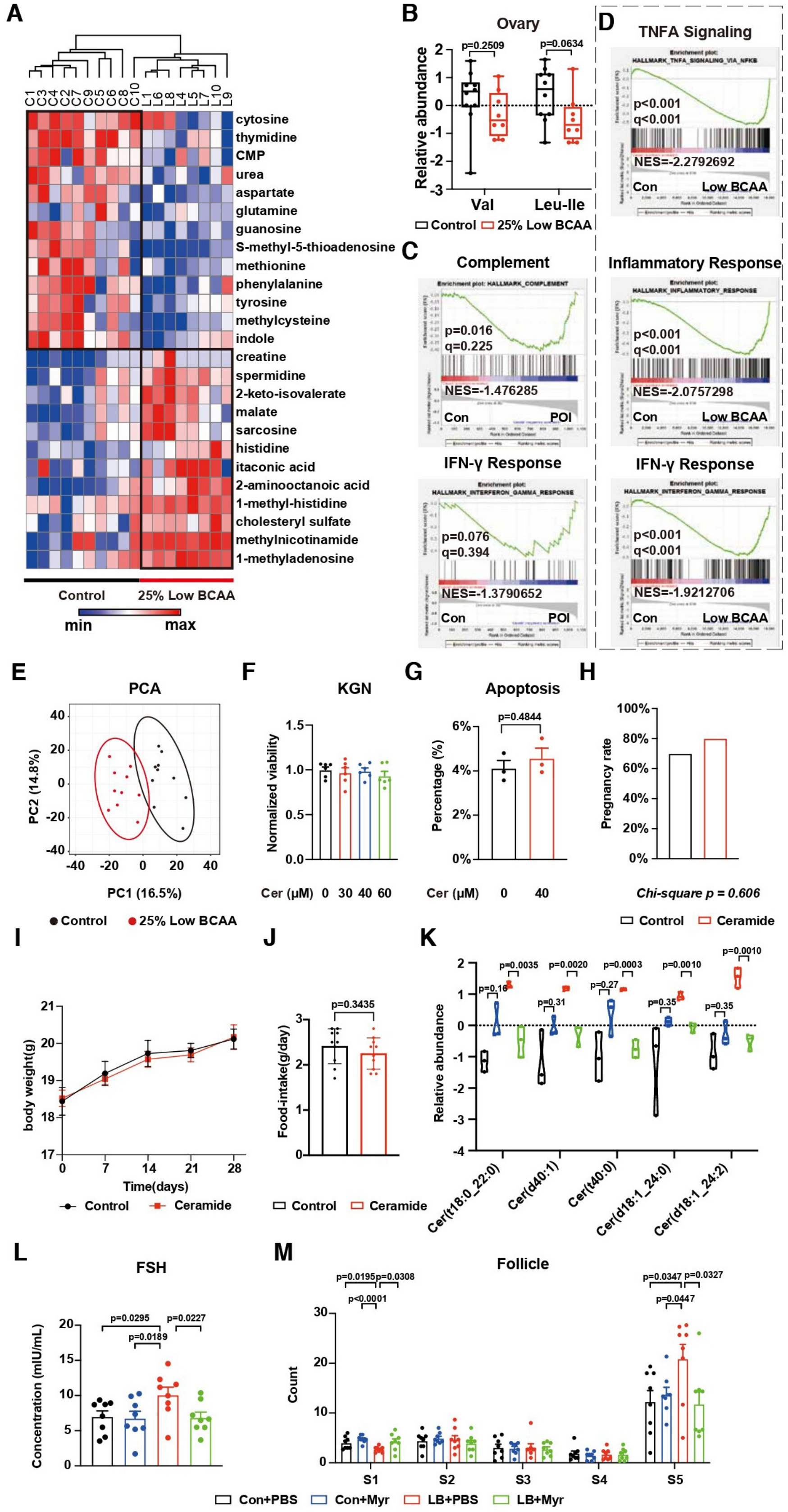

Figure EV2. Lipid metabolism features of POI.

A Heatmap showing the top 25 differentially abundant metabolites in the ovaries of mice on a low BCAA diet. Control, $N = 10$; low BCAA, $N = 8$.

B The relative abundance of BCAAs in the ovaries of mice on a low BCAA diet. Control, $N = 10$; low BCAA, $N = 8$; Boxplot, central band stands for median, boxes stand for 50% of the data, and whiskers stand for min or max of the data.

C GSEA of the proteomics data in the serum of the Fudan Cohort. $N = 11$.

D GSEA of RNA-seq data from the liver in the mice on a low BCAA diet or control diet. $N = 6$.

E The PCA of serum from mice on a low BCAA diet or control diet. $N = 10$.

F The relative cell viability of KGN cells with ceramide treatment. $N = 6$.

G The percentage of Annexin V positive cells of KGN cells with ceramide treatment. $N = 3$.

H–J The pregnancy rate, body weight, and food intake from mice with ceramide treatment. $N = 10$.

K The relative abundance of ceramide in the serum of mice with myriocin treatment. $N = 3$; Truncated violin plot, central band stands for median, and dotted lines stand for the upper quartile or the lower quartile of the data.

L The serum concentration of FSH in mice with ceramide treatment. $N = 8$.

M The changes in follicle count from mice with ceramide treatment. $N = 8$.

Data information: S1, Primordial; S2, Primary; S3, Secondary; S4, Antral; S5, Atretic. Error bars stand for SEM. The *P*-value was calculated by a two-tailed *t*-test with 2-way ANOVA correction.
Source data are available online for this figure.

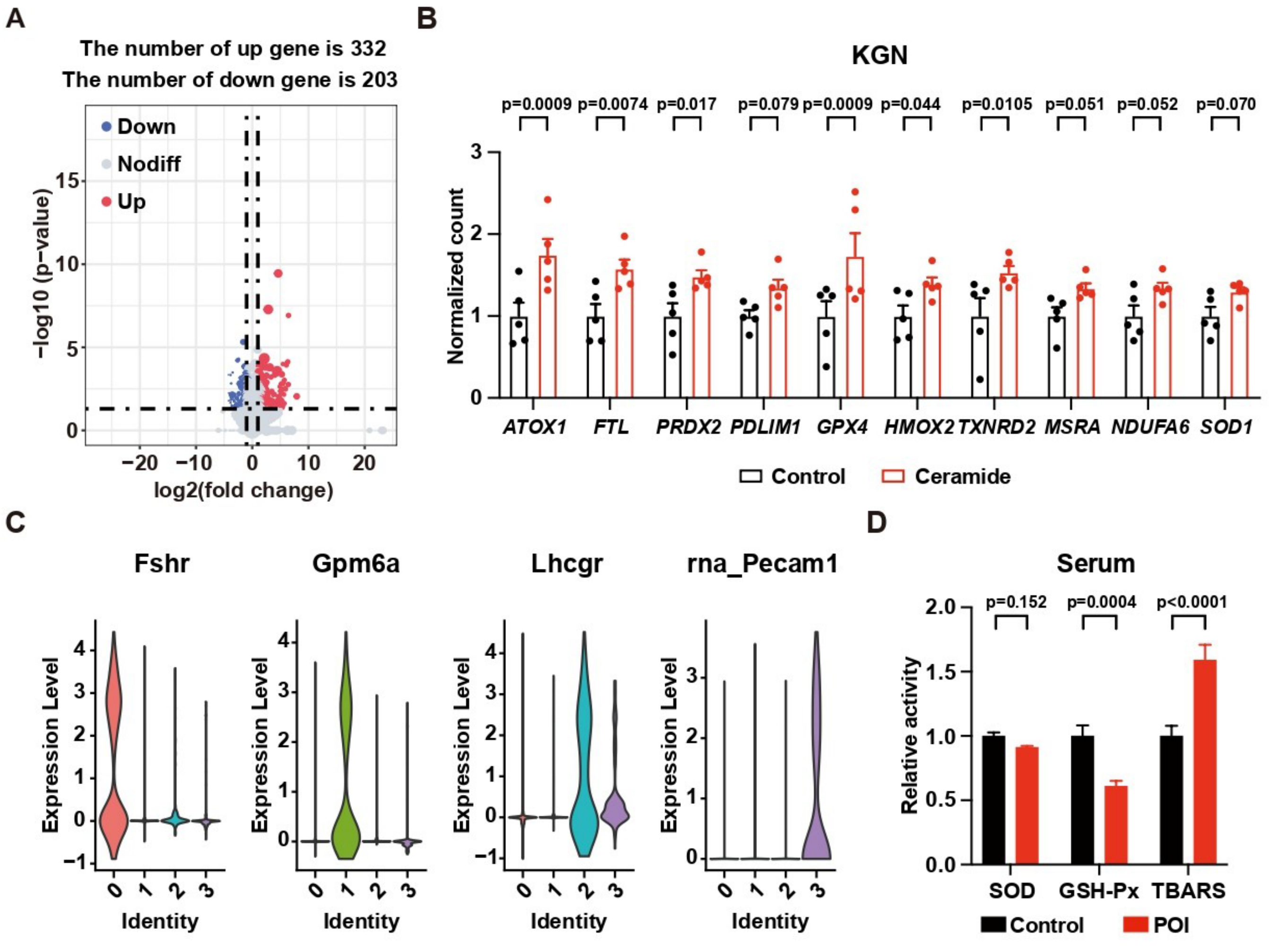

Figure EV3. Low BCAA induces POI via elevation of ROS.

A Genes with two-fold changes and *P*-value < 0.05 are highlighted in the volcano plot. *N* = 5.
B Upregulation of ROS-related genes in KGN cells treated with ceramide. *N* = 5.
C Violin plots showing the expression of classical markers of nonimmune cells in ovaries from sNuc-seq data.
D Relative activity of ROS-related factors in patients. Control, *N* = 30; POI, *N* = 60.

Data information: Error bars stand for SEM. The *P*-value was calculated by a two-tailed *t*-test with 2-way ANOVA correction.
Source data are available online for this figure.

© 2023 The Authors

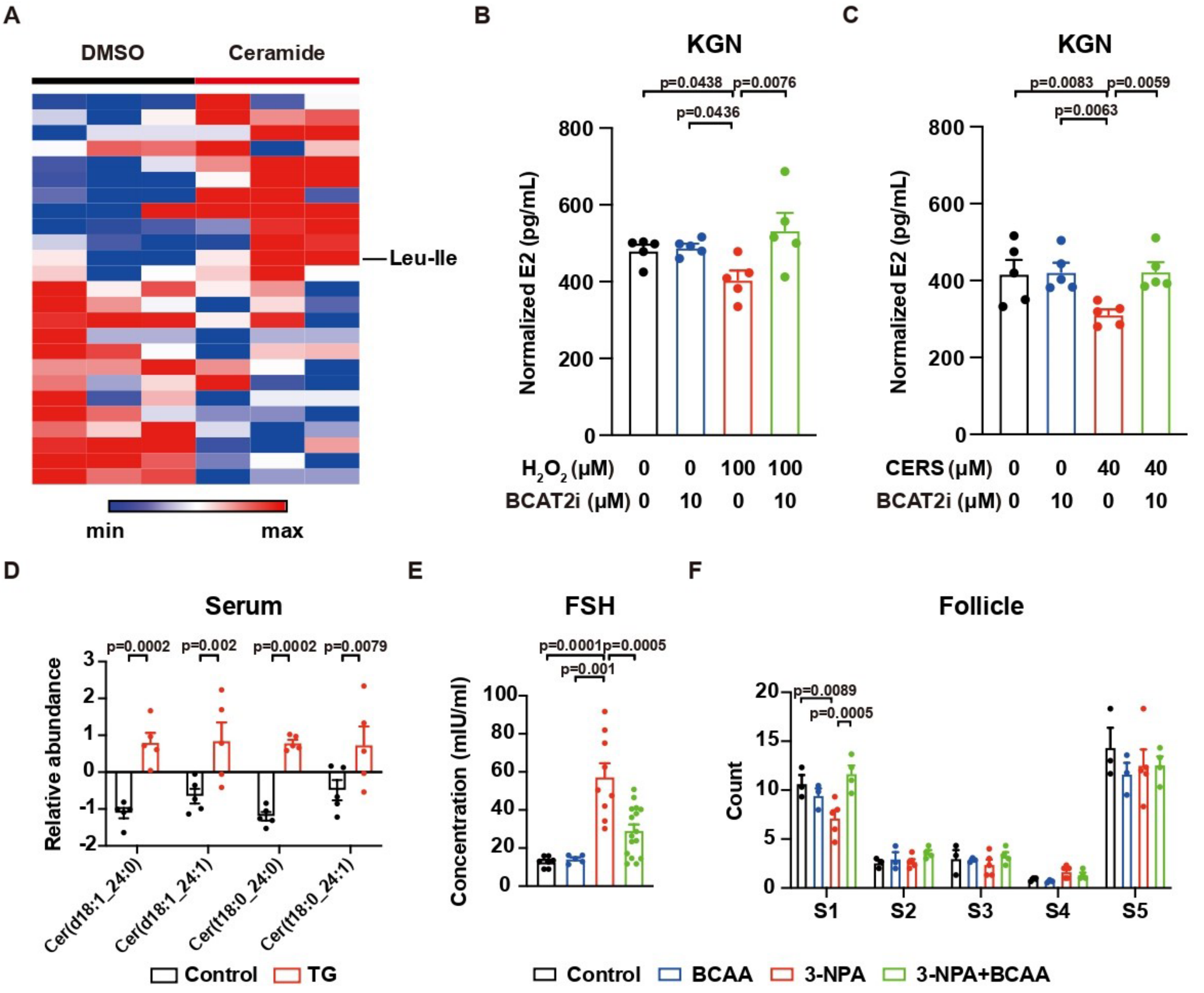

Figure EV4. BCAA supplement protected the granulosa cells from ROS inducer.

A Heatmap showing the relative abundance of metabolites in KGN cells treated with ceramides. *N* = 3.

B The concentration of E2 secreted by KGN cells with H_2O_2 treatment w/o BCAT2 inhibitor. *N* = 5.

C The concentration of E2 secreted by KGN cells with ceramide treatment w/o BCAT2 inhibitor. *N* = 5.

D Elevation of ceramide in the serum of TG-treated mice. *N* = 5.

E BCAA supplement prevented the elevation of FSH in mice with 3-NPA treatment. Control, *N* = 7; BCAA, *N* = 5; 3-NPA, *N* = 9; 3-NPA + BCAA, *N* = 16.

F BCAA supplement rescued the decrease of primordial follicles in mice with 3-NPA treatment. Control, *N* = 3; BCAA, *N* = 3; 3-NPA, *N* = 5; 3-NPA + BCAA, *N* = 4.

Data information: S1, Primordial; S2, Primary; S3, Secondary; S4, Antral; S5, Atretic. Error bars stand for SEM. The *P*-value was calculated by a two-tailed *t*-test with 2-way ANOVA correction.
Source data are available online for this figure.

Appendix Figures

Appendix FigureS1 2
Appendix FigureS2 3
Appendix FigureS3 4
Appendix FigureS4 5
Appendix FigureS5 5

Appendix Figure

A

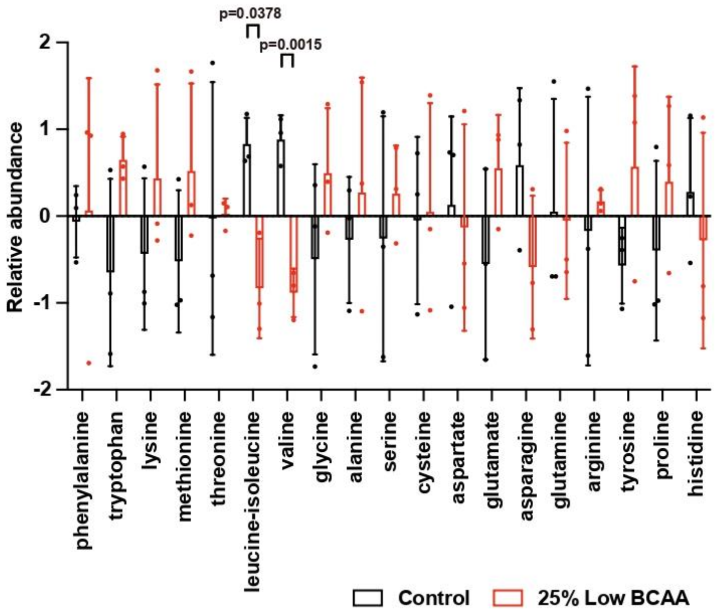

B

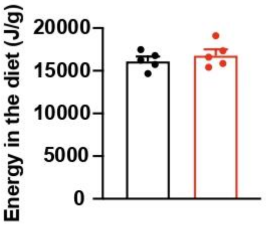

C

Ingredient (g)	Control	50% BCAA	25%BCAA
Amino acid mixture without BCAA	151	151	151
BCAA mixture	28	14	7
Corn starch	417	431	437
Dextrin and sucrose	250	250	250
Soy oil	50	50	50
Cellulose	50	50	50
Mineral mixture	43	43	43
Vitamin mixture	10	10	10
Choline chloride	2	2	2
TBHQ, antioxidant	0.01	0.01	0.01
Total	1000	1000	1000

D

Ingredient (g)	Control	25%BCAA
L-Arginine	10	10
L-Histidine-HCl-H2O	6	6
L-isoleucine	8	2
L-Leucine	12	2
L-Lysine-HCl	14	14
L-Methionine	6	6
L-Phenylalanine	8	8
L-Threonine	8	8
L-Typtophan	2	2
L-Valine	8	2
L-Alanine	10	10
L-Asparagine-H2O	5	5
L-Aspartate	10	10
L-Cystine	4	4
L-Glutamic Acid	30	30
L-Glutamine	5	5
Glycine	10	10
L-Proline	5	5
L-Serine	5	5
L-Tyrosine	4	4
Corn Starch	550.5	572.5
Maltodextrin 10	125	125
Cellulose	50	50
Corn Oil	50	50
Mineral Mix S10001	35	35
Sodium Bicarbonate	7.5	7.5
Vitamin Mix V10001	10	10
Choline Bitrartrate	2	2
FD&C Blue Dye #1	0.05	0.05
Total	1000.05	1000.05

Appendix FigureS1. Low BCAA diet on young female mice.

A. The relative abundance of amino acid composition of the low BCAA diet. N=3; **B.** The energy in the diet. N=5; and **C.** The recipe of the diet from Trophic Animal Feed High-tech; **D.** The recipe of the diet from Research Diet, Inc. Error bars stand for SEM of biological repeats. The p value was calculated by two-tailed t-test with 2-way ANOVA correction.

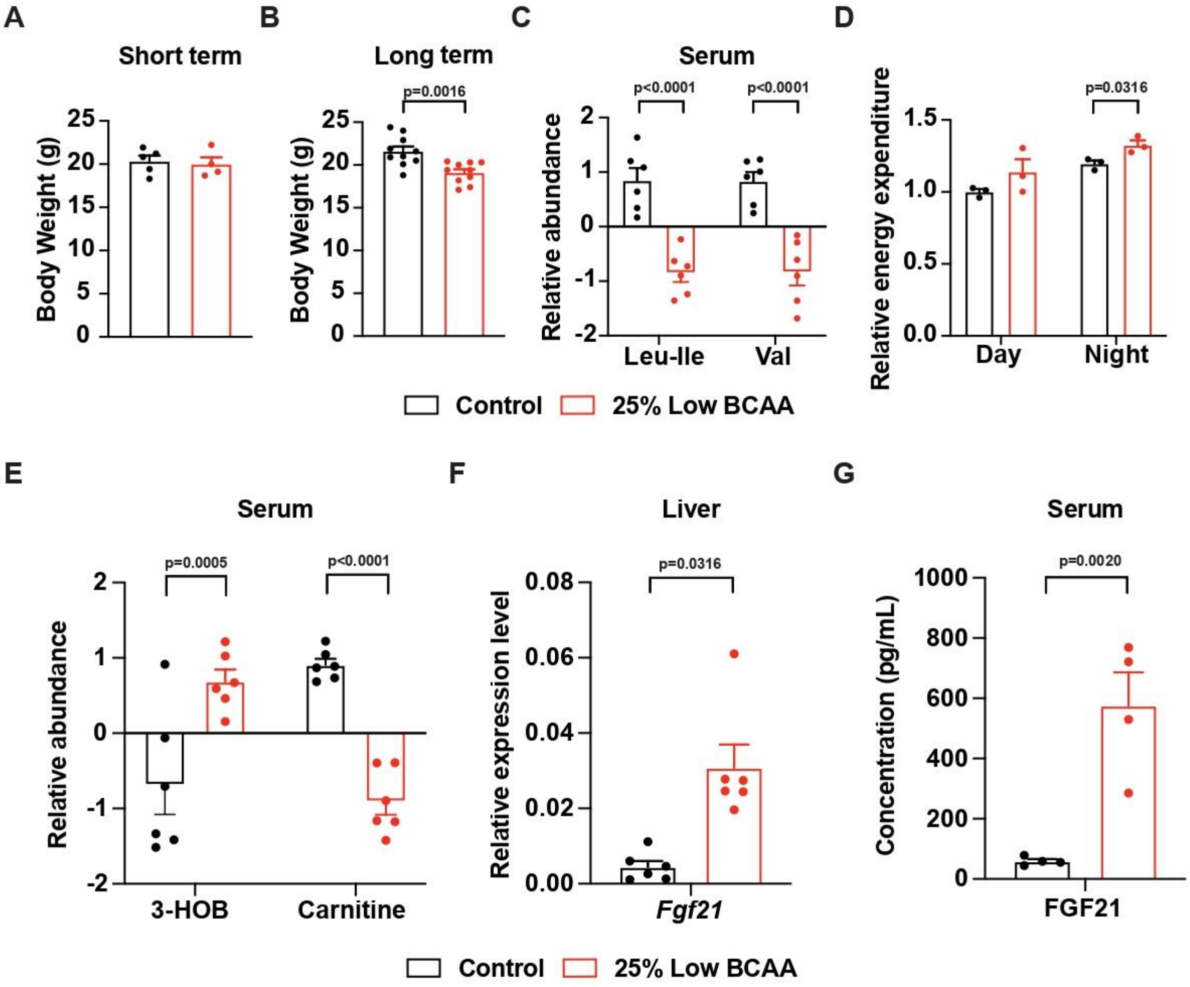

Appendix FigureS2. Metabolic features of young female mice fed a low BCAA diet.

A and B. The body weight of short (1.5 months; Control, N=5; Low BCAA, N=4) and long (3 months, N=10) term low BCAA diet; **C.** The relative abundance of BCAAs in serum. N=6; **D.** The relative energy expenditure measured by Comprehensive Lab Animal Monitoring System (CLAMS). N=3; **E.** The relative abundance of 3-hydroxybutyrate (3-HOB) and carnitine in serum. N=6; **F.** The mRNA level of Fgf21 in liver. N=6; **G.** The concentration of FGF21 in serum. N=4. Error bars stand for SEM of biological repeats. The p value was calculated by two-tailed t-test with 2-way ANOVA correction.

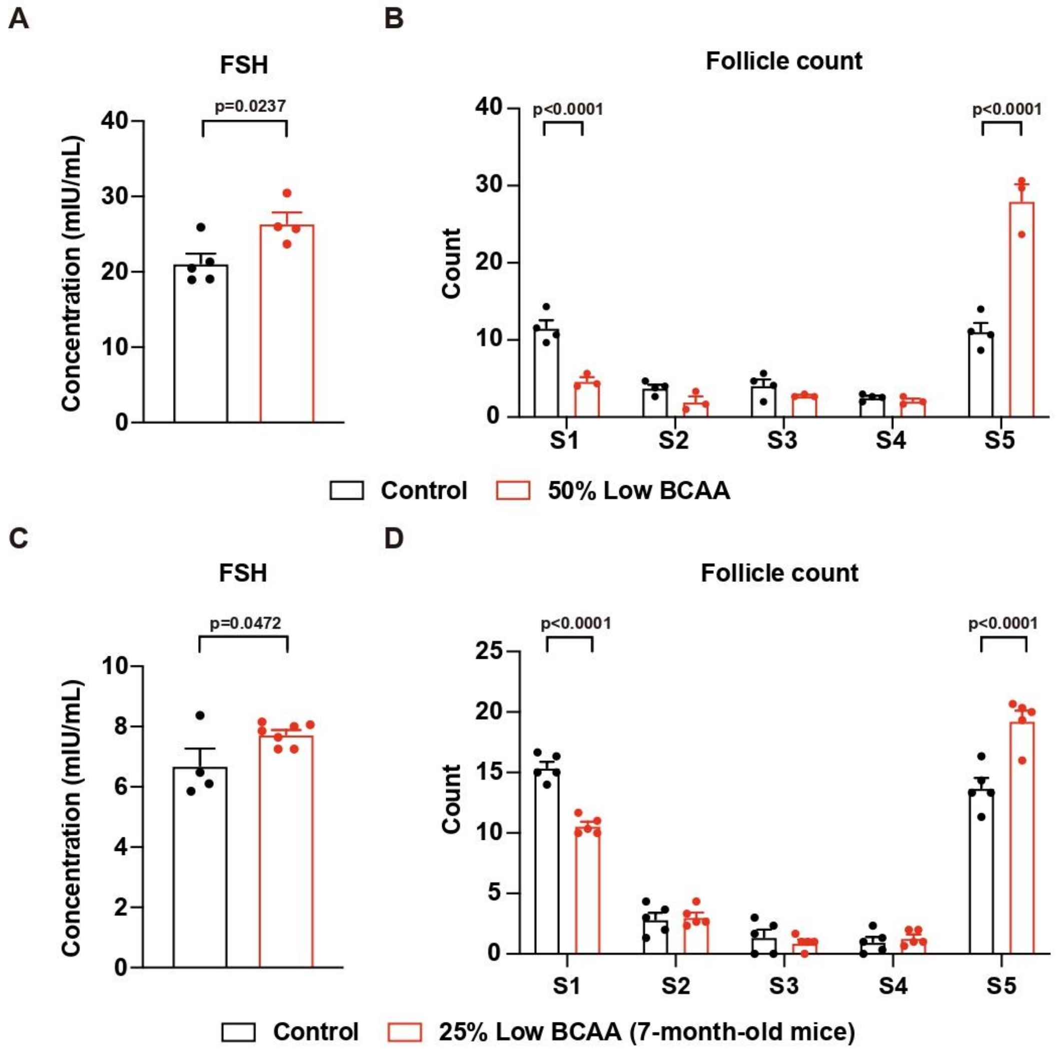

Appendix FigureS3. BCAA insufficiencies induce POI.

A. The serum concentration of FSH. Control, N=5; Low BCAA, N=4; and **B.** The changes of follicles in the mice on 50% low BCAA diet. Control, N=4; Low BCAA, N=3; **C.** Serum concentration of FSH. Control, N=4; Low BCAA, N=7; and **D.** The changes of follicles in 7-month-old mice on 25% low BCAA diet. N=5. S1, Primordial; S2, Primary; S3, Secondary; S4, Antral; S5, Atretic. Error bars stand for SEM of biological repeats. The p value was calculated by two-tailed t-test with 2-way ANOVA correction.

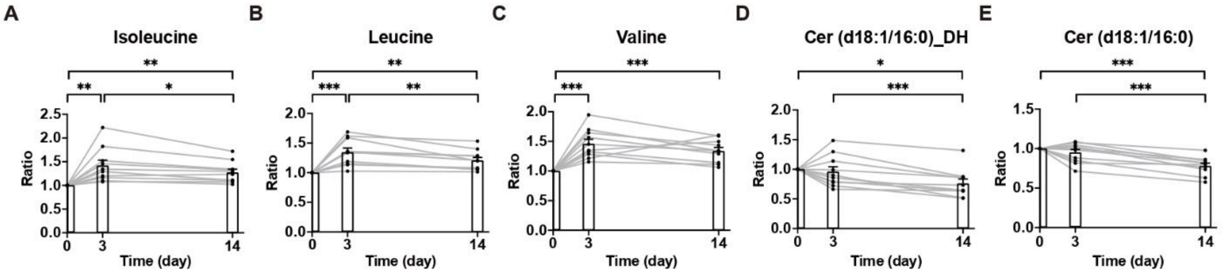

Appendix FigureS4. Relative abundance of metabolites in the serum of patients on a low carbohydrate high protein diet.

A. Serum concentration of isoleucine. N=7; **B.** Serum concentration of leucine. N=7; **C.** Serum concentration of valine. N=7; **D.** Serum concentration of cer (d18:1/16:0)_DH. N=7; **E.** Serum concentration of cer (d18:1/16:0). N=7. Error bars stand for SEM of biological repeats. The p value was calculated by two-tailed t-test with 2-way ANOVA correction.

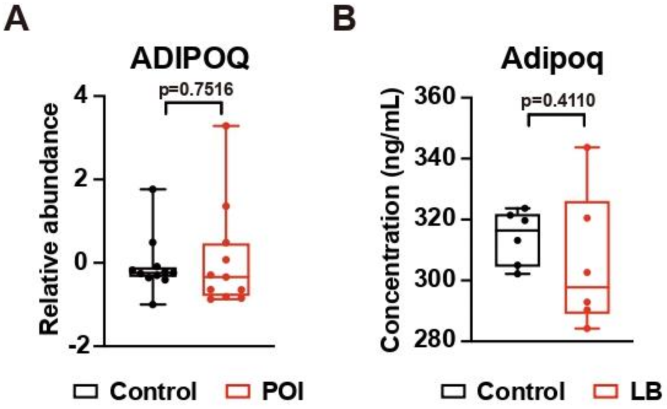

Appendix FigureS5. The changes of adiponectin in POI patients and mice on a low BCAA diet.

A. Serum concentration of adiponectin in POI patients and healthy donors. N=18; **B.** Serum concentration of adiponectin in mice fed by low BCAA diet (LB) or control diet. N=6. Error bars stand for SEM of biological repeats.
